# Epidemiology of Hospital Admissions with Influenza during the 2013/2014 Northern Hemisphere Influenza Season: Results from the Global Influenza Hospital Surveillance Network

**DOI:** 10.1371/journal.pone.0154970

**Published:** 2016-05-19

**Authors:** Joan Puig-Barberà, Angels Natividad-Sancho, Svetlana Trushakova, Anna Sominina, Maria Pisareva, Meral A. Ciblak, Selim Badur, Hongjie Yu, Benjamin J. Cowling, Clotilde El Guerche-Séblain, Ainara Mira-Iglesias, Lidiya Kisteneva, Kirill Stolyarov, Kubra Yurtcu, Luzhao Feng, Xavier López-Labrador, Elena Burtseva, Global Influenza Hospital Surveillance Study Group (GIHSN)

**Affiliations:** 1 Fundación para el Fomento de la Investigación Sanitaria y Biomédica de la Comunidad Valenciana (FISABIO), Valencia, Spain; 2 D.I. Ivanovsky Institute of Virology FSBI “N.F. Gamaleya FRCEM” Ministry of Health of Russian Federation, Moscow, Russian Federation; 3 Research Institute of Influenza, St. Petersburg, Russian Federation; 4 National Influenza Reference Laboratory, Istanbul Faculty of Medicine, Istanbul University, Istanbul, Turkey; 5 Division of Infectious Disease, Key Laboratory of Surveillance and Early-Warning on Infectious Disease, Chinese Center for Disease Control and Prevention, Beijing, China; 6 School of Public Health, Li Ka Shing Faculty of Medicine, The University of Hong Kong, Hong Kong Special Administrative Region, China; 7 Sanofi Pasteur, Lyon, France; 8 Centro de Investigación Biomédica en Epidemiología y Salud Publica (CIBER-ESP), Instituto de Salud Carlos III, Madrid, Spain; University of Waterloo, CANADA

## Abstract

**Background:**

The Global Influenza Hospital Surveillance Network was established in 2012 to obtain valid epidemiologic data on hospital admissions with influenza-like illness. Here we describe the epidemiology of admissions with influenza within the Northern Hemisphere sites during the 2013/2014 influenza season, identify risk factors for severe outcomes and complications, and assess the impact of different influenza viruses on clinically relevant outcomes in at-risk populations.

**Methods:**

Eligible consecutive admissions were screened for inclusion at 19 hospitals in Russia, Turkey, China, and Spain using a prospective, active surveillance approach. Patients that fulfilled a common case definition were enrolled and epidemiological data were collected. Risk factors for hospitalization with laboratory-confirmed influenza were identified by multivariable logistic regression.

**Findings:**

5303 of 9507 consecutive admissions were included in the analysis. Of these, 1086 were influenza positive (534 A(H3N2), 362 A(H1N1), 130 B/Yamagata lineage, 3 B/Victoria lineage, 40 untyped A, and 18 untyped B). The risk of hospitalization with influenza (adjusted odds ratio [95% confidence interval]) was elevated for patients with cardiovascular disease (1.63 [1.33–2.02]), asthma (2.25 [1.67–3.03]), immunosuppression (2.25 [1.23–4.11]), renal disease (2.11 [1.48–3.01]), liver disease (1.94 [1.18–3.19], autoimmune disease (2.97 [1.58–5.59]), and pregnancy (3.84 [2.48–5.94]). Patients without comorbidities accounted for 60% of admissions with influenza. The need for intensive care or in-hospital death was not significantly different between patients with or without influenza. Influenza vaccination was associated with a lower risk of confirmed influenza (adjusted odds ratio = 0.61 [0.48–0.77]).

**Conclusions:**

Influenza infection was detected among hospital admissions with and without known risk factors. Pregnancy and underlying comorbidity increased the risk of detecting influenza virus in patients hospitalized with influenza-like illness. Our results support influenza vaccination as a measure for reducing the risk of influenza-associated hospital admission.

## Introduction

Influenza is a significant local and global public health problem that causes substantial year-round morbidity and mortality [1]. Several surveillance systems monitor influenza disease activity with the aim of better understanding its epidemiology and the impact of control measures [2–7]. However, current surveillance systems suffer from several limitations, including non-systematic sampling, incomplete case ascertainment, lack of adjustment for confounders, scarcity of information on the impact of different influenza viruses, sparse numbers, lack of consensus about case definition and risk factors, and a lack of comparison groups [8–10]. Also, the value of the resulting analyses is often limited by a lack of adjustment for confounders and relatively small sample sizes. These limitations are especially notable for the hospital setting, even though severe influenza is one of the most influential health economic parameters in cost-effectiveness calculations [9,11,12].

Identifying risk factors for severe outcomes and complications is important for reducing influenza-related morbidity and mortality and for guiding control measures against influenza. To assess the impact of the different influenza viruses on clinically meaningful outcomes in at-risk populations, extensive data are needed from geographically diverse settings over several influenza seasons and collected using a common core protocol.

The Global Influenza Hospital Surveillance Network (GIHSN) was established in 2012 as a public-private partnership to obtain valid epidemiologic data on influenza admissions, with the objective of informing influenza prevention and control policies. The GIHSN, which included hospitals in Russia, China, Turkey, and Spain during the 2013/2014 influenza season [6], uses a common core protocol to promote consistent eligibility criteria, case definition, and systematic swabbing. Consistency of the information collected is further facilitated by using reverse transcription-polymerase chain reaction (RT-PCR) to confirm influenza infection, by following standard operating procedures, and by using a shared core questionnaire to collect patient information [6,13].

Here, we describe the epidemiology of hospital admissions with influenza during the 2013/2014 influenza season in the GIHSN Northern hemisphere participating sites. We also determine the impact of underlying patient characteristics on the risk of hospital admission and complications due to influenza overall and due to influenza A(H1N1)pdm09, A(H3N2), and B/Yamagata lineage.

## Methods

### Study design and participants

This study employed a prospective active surveillance approach to collect epidemiological and virological data for the 2013/2014 Northern hemisphere influenza season. The participating sites included four hospitals in the Russian Federation, seven in Turkey, two in China, and six in Spain (S1 Table).

The methods used in this study were described previously [6,13,14]. Briefly, eligible admissions included non-institutionalized residents in the predefined catchment areas of the participating hospitals, hospitalized in the last 48 h, and with presenting complaints potentially associated with influenza (S2 and S3 Tables). The study activities were performed over influenza circulation periods defined using pre-specified criteria (S3 Table). The study protocol was approved by the institutional review board of each participating site, Comité Ético de la Dirección General de Salud Pública y Centro Superior de Investigación en Salud Pública (CEIC-DGSP-CSISP); Ethical Committee of Hospital #1 for Infectious Diseases of Moscow Health Department; Ethics Committee of the Research Institute of Influenza, St. Petersburg; Istanbul University, Istanbul Faculty of Medicine, Ethical Committee for Clinical Research. The study was carried in China by the Chinese Center for Disease Control and Prevention, Beijing, China as part of the implementation of the national Severe Acute Respiratory Infections (SARI) surveillance program in China for purposes of communicable disease control. Ethical approval was not required but informed oral consent was sought before inclusion. All subjects or legal representatives provided written or oral informed consent.

### Inclusion criteria

Eligible admissions were approached by healthcare professionals trained to follow the GIHSN generic study protocol (S2 and S3 Tables). All sites applied the same selection criteria to eligible admissions. Patients ≥5 years of age had to have influenza like illness (ILI) symptoms [15], defined as at least one systemic symptom (fever or feverishness, malaise, headache, or myalgia) and at least one respiratory symptom (cough, sore throat, or shortness of breath), and hospitalization within 7 days of the onset of ILI. Patients <5 years of age had to be admitted to hospital within 7 days of the appearance of symptoms potentially associated with influenza. Patients who had been discharged from a hospital within 30 days of the current episode were excluded.

### Sampling

After patients or legal representatives provided signed, informed consent, a combined nasopharyngeal and pharyngeal swab (patients ≥14 years of age) or both nasopharyngeal and nasal swabs (patients <14 years old) were obtained from included subjects in the Russian Federation, Turkey, and Spain. In China, two combined pharyngeal swabs were obtained from all patients regardless of age. Both swabs were collected in the same test tube and sent to the site’s laboratory for testing (S1 Text).

### Covariates

Demographic, clinical, and epidemiological information was collected from all enrolled patients as previously described [6,13]. Baseline data collected included date of symptom onset, date of admission, sex, age, presence of chronic conditions, pregnancy status, obesity, smoking habits, hospital admissions in the past 12 months, outpatient consultations in the previous 3 months, socioeconomic status based on occupation [16], functional status in patients ≥65 years of age (Barthel index) [17], and time elapsed between illness onset and swabbing. Vaccination in the previous and current season was ascertained by recall, clinical records, or vaccination registries. Date of discharge or death while hospitalized, intensive care unit (ICU) admission, and main diagnoses at discharge or at time of death were also recorded.

Obesity was defined as a body mass index ≥30 kg/m^2^ for subjects ≥18 years of age. For subjects <18 years of age, obesity criteria were applied following World Health Organization guidelines [18,19].

### Laboratory methods

Influenza infection was confirmed by RT-PCR for influenza A (subtypes H3 and H1pdm09) and B (Yamagata and Victoria lineages) (S1 Text).

### Outcomes

Admissions with a positive RT-PCR result were considered influenza-related admissions. The distribution of hospital admission according to RT-PCR result was described by site and risk group. Secondary outcomes included hospital admissions by subtype for influenza A(H1N1)pdm09, A(H3N2), and B/Yamagata lineage, by site and risk group.

### Data analysis

Statistical analyses were performed using Stata version 14.0 (College Station, TX). All analyses were restricted to periods with influenza admissions. Records with missing laboratory results or date of illness onset were excluded. Only admissions swabbed within 7 days after symptom onset were included in the analysis. The significance of differences among groups or categories was estimated by the likelihood ratio test, t-test, or nonparametric tests as required. Additional analyses included the number and temporal sequence of admissions and the distribution of type subtypes of viruses, sociodemographic characteristics, underlying conditions and exposures, ICU admission, length of stay, in-hospital death, major complications, and main diagnoses at discharge [20].

### Risk for influenza related admission

To describe the major determinants for admission with influenza (vs. influenza-negative admission), a stepwise logistic regression model was fitted by including all risk factors at P<0.2.

### Confounding and adjusted estimates

Adjusted odds ratios (aORs) for RT-PCR-positive vs. RT-PCR-negative admissions in the presence of major risk factors of interest were estimated by multivariate logistic regression using minimal sufficient adjustment sets of covariates identified as confounders by causal diagrams [21,22]. When more than one alternative for modeling was possible, we used the most explanative and parsimonious model according to Akaike’s information criterion [23]. To estimate risk factors for specific strains, admissions positive for other influenza strains were excluded. When comparing the effects of comorbidities, the comparison group was individuals without underlying conditions. For pregnant women, the comparison group was women 15 to 45 years of age. For variables with more than one category, the lowest was used as a baseline for comparison. Restricted cubic spline functions were used to model the effect of age and calendar on influenza-associated events, with the best modeling (number of knots) option decided using Akaike’s information criterion [23]. The likelihood ratio test was used to estimate significance and to explore linearity and effect modification.

### Clustering

To account for the possible effect of study site, data were fitted to a random effects logistic regression model including site as a cluster variable. Likelihood ratio tests were used to check for the potential effect of clustering by site [24]. The adjusted effect of site in the probability of influenza with admission was estimated.

### Heterogeneity

Heterogeneity in the effects of risk factors by influenza strain and site were quantified using the I^2^ test. Heterogeneity was defined as an I^2^>50% [25,26]. Forest plots were used to present the effect size of each risk factor by strain.

### Sample size

Assuming that 20% of admissions in the control group are due to influenza and with a type II error <20% and a type I error ≤5% [27], 1825 patients are needed to detect a significant impact of a comorbid condition at an odds ratio of 1.25, 525 at an odds ratio of 1.50, and 175 at an odds ratio of 2.0.

## Results

### Included patients

Between December 2013 and June 2014, 9507 eligible admissions were identified by the 19 participating hospitals. Of these, 5303 (56%) were included and swabbed (Table 1). Major reasons for exclusion were the absence of ILI symptoms within 7 days of admission (n = 1781 [19%]), swabbing more than 7 days after symptoms onset (n = 1006 [11%]), not obtaining informed consent (n = 591 [6%]), and recruitment during periods without influenza admissions (n = 279 [3%]) (Table 1).

**Table 1:** Flow of study patients and RT-PCR results.

	St. Petersburg		Moscow		Turkey		Beijing		Valencia		Total	
	N = 1713		N = 1743		N = 1509		N = 655		N = 3887		N = 9507	
Category	n	%	n	%	n	%	n	%	n	%	n	%
Excluded from the analysis												
Nonresident	11	0.6	63	3.61	127	8.4	1	0.2	49	1.3	251	2.6
Institutionalized	5	0.3	11	0.63	1	0.1	5	0.8	11	0.3	33	0.3
Unable to communicate	28	1.6	47	2.70	21	1.4	8	1.2	175	4.5	279	2.9
Did note provide consent	74	4.3	59	3.38	39	2.6	69	10.5	71	1.8	312	3.3
Previous discharge from hospital <30 days	13	0.8	44	2.52	152	10.1	8	1.2	27	0.7	244	2.6
No ILI symptoms, ≥5 years of age	4	0.2	39	2.24	558	37.0	40	6.1	1140	29.3	1781	18.7
Swabbed >7 days after onset of symptoms (all ages)	286	16.7	149	8.55	103	6.8	61	9.3	407	10.5	1006	10.6
Sample inadequate	0	0.0	0	0.00	0	0.0	0	0.0	5	0.1	5	0.1
Previous influenza infection	1	0.1	1	0.06	9	0.6	0	0.0	3	0.1	14	0.1
Recruited in weeks without laboratory-confirmed Influenza cases	35	2.0	70	4.02	13	0.9	42	6.4	119	3.1	279	2.9
Included in the analysis	1256	73.3	1260	72.29	486	32.2	421	64.3	1880	48.4	5303	55.8
RT-PCR result												
Negative	1026	81.7	979	77.7	372	76.5	331	78.6	1509	80.3	4217	79.5
Influenza positive	230^a^	18.3	281	22.3	114	23.5	90	21.4	371	19.7	1086	20.5
Influenza A(H1N1)	25	2.0	27	2.1	0	0.0	15	3.6	295	15.7	362	6.8
Influenza A(H3N2)	156	12.4	186	14.8	90	18.5	35	8.3	67	3.6	534	10.1
Influenza A/not typed	23	1.8	7	0.6	0	0.0	1	0.2	9	0.5	40	0.8
Influenza B/Yamagata	15	1.2	52	4.1	24	4.9	39	9.3	0	0.0	130	2.5
Influenza B/Victoria	0	0.0	3	0.2	0	0.0	0	0.0	0	0.0	3	0.1
Influenza B/not typed	12	1.0	6	0.5	0	0.0	0	0.0	0	0.0	18	0.3

^a^ The number of influenza-positive subjects does not match the sum of the individual strains because one subject was infected with both A(H1N1) and A(H3N2) influenza.

**Fig 1:**
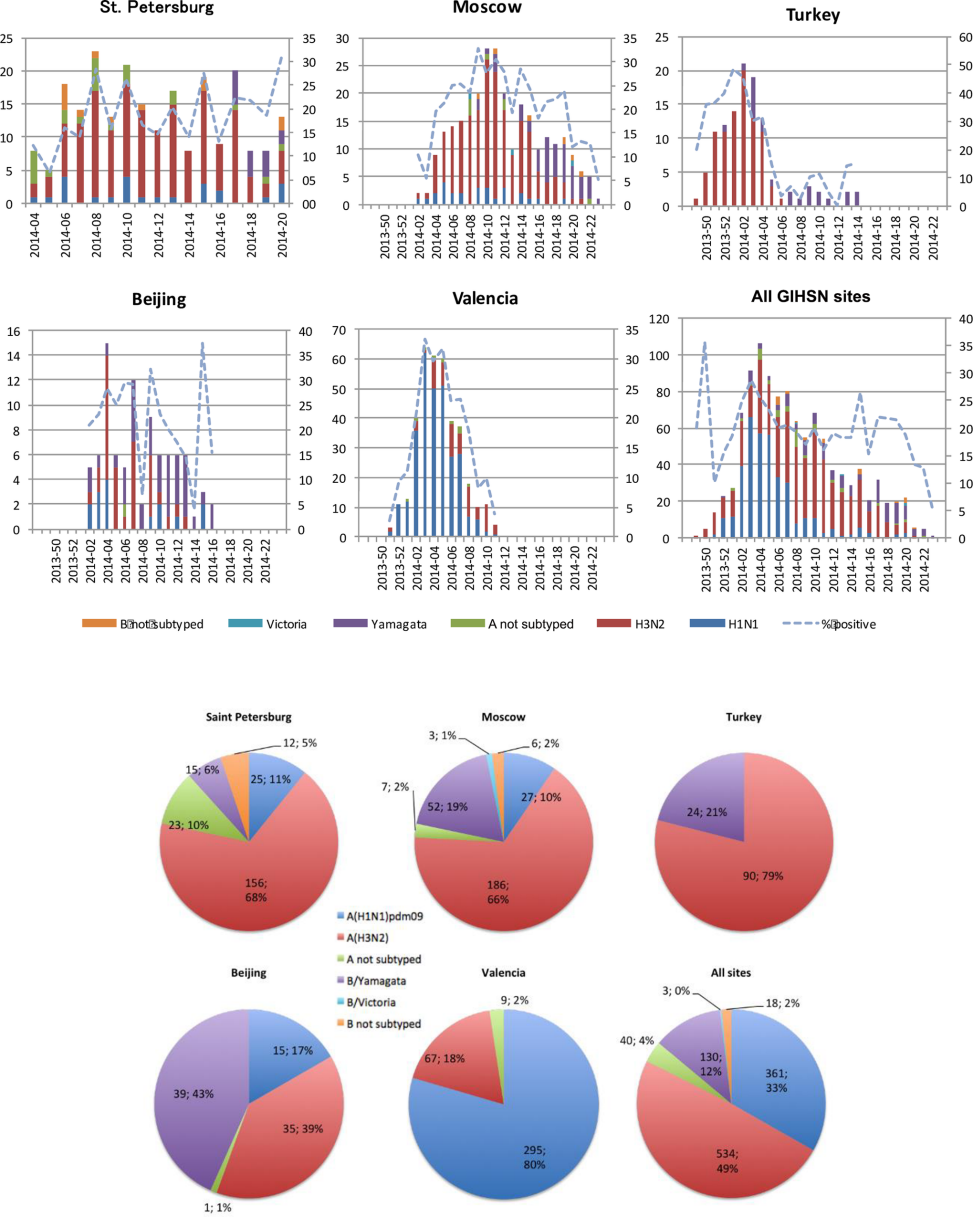
Admissions with influenza by site, by epidemiological week, and by influenza virus type, subtype, or lineage.

Of the 5303 admissions, 1086 (21%) had laboratory-confirmed influenza (Table 1, Fig 1). The lowest percentage of influenza positivity was in St. Petersburg (18%) and the highest was in Turkey (24%). Ascertainment of positive admission was earlier in Turkey (week 49/2013) and Valencia (week 51/2013) than in Moscow (week 2/2014), Beijing (week 2/2014), or St. Petersburg (4/2014). The number of consecutive weeks with influenza-positive admissions ranged from 13 to 21 (13 in Valencia, 14 in Beijing, 17 in St. Petersburg and Turkey, and 21 in Moscow).

### Circulating influenza strains

Overall, influenza A(H3N2) was the predominant circulating strain (n = 543, 50%), followed by A(H1N1)pdm09 (n = 362; 34%) and B/Yamagata lineage (n = 130; 12%) (Table 1, Table 2 and Fig 1). Only three admissions had B/Victoria lineage in Moscow. At each site, the influenza viruses detected varied during the season.

**Table 2:** Characteristics of included patients according to RT-PCR result.

	Influenza negative		Influenza positive		A(H3N2)^a^		A(H1N1)p^a^		B/Yamagata^a^	
	**N = 4217**		**N = 1086**		**N = 534**		**N = 362**		**N = 130**	
Characteristic	n	%	n	%	n	%	n	%	n	%
Age in years, mean (standard deviation)	30.9 (31.7)		34.9 (28.6)		30 (27.0)		45.8* (29.3)		30.6 (25.6)	
Age group										
0–5 months	503	11.9	54	5	22	4.1	21	5.8	3	2.3
6–11 months	279	6.6	36	3.3	20	3.7	11	3	1	0.8
1–4 years	905	21.5	181	16.7	100	18.7	44	12.2	23	17.7
5–17 years	291	6.9	102	9.4	57	10.7	14	3.9	24	18.5
18–49 years	929	22.0	349	32.1	214	40.1	70	19.4	49	37.7
50–64 years	337	8.0	132	12.2	29	5.4	87	24.1	11	8.5
65–74 years	304	7.2	94	8.7	32	6.0	51	14.1	8	6.2
75–84 years	426	10.1	106	9.8	47	8.8	47	13	9	6.9
≥85 y	243	5.8	32	2.9	13	2.4	16	4.4	2	1.5
Female	1829	43.4	547	50.4*	284	53.2*	165	45.7	72	55.4*
Number of underlying comorbidities										
0	2789	66.1	656	60.4	345	64.6	165	45.7	94	72.3
1	732	17.4	260	23.9*	122	22.8*	109	30.2*	24	18.5
≥2	696	16.5	170	15.7	67	12.5	87	24.1*	12	9.2
Underlying chronic diseases										
Cardiovascular disease	685	17.1	189	18.4	91	17.9	80	22.8*	13	12.3
COPD	607	15.2	139	13.6	44	8.7*	79	22.5*	13	12.3
Asthma	194	4.8	76	7.4*	35	6.9	31	8.8*	10	9.4
Immunodeficiency/organ transplant	38	0.9	16	1.6	13	2.6*	3	0.9	0	0
Diabetes	390	9.7	105	10.2	30	5.9*	67	19.1*	5	4.7
Chronic renal impairment	167	4.2	57	5.6	24	4.7	28	8*	4	3.8
Chronic rheumatologic disease	12	0.5	11	1.7*	6	1.4	1	1.8	4	3.8*
Chronic neuromuscular disease	94	2.3	19	1.9	14	2.8	3	0.9*	2	1.9
Active neoplasm	130	3.2	29	2.8	13	2.6	15	4.3	0	0
Chronic liver disease	65	1.6	23	2.2	10	2.0	11	3.1	1	0.9
Autoimmune disease	32	0.8	16	1.6*	8	1.6	6	1.7	2	1.9
Pregnant status (women 15–45 years)	181	44.7	156	70.3*	100	71.9*	18	51.4.	33	86.8*
Obese^b^	701	16.9	194	18.1	77	14.6	95	26.5*	14	10.9
Outpatient visits in the last 3 months^c^										
0	1283	30.5	306	28.2	172	32.2	85	23.6	30	23.1
1	919	21.8	241	22.2	93	17.4	112	31.1	19	14.6
≥2	2011	47.7	538	49.6	269	50.4	163	45.3	81	62.3*
Smoking habits^d^										
Current smoker	1009	23.9	274	25.3	124	23.2	100	27.7	31	24
Past smoker	813	19.3	200	18.4	96	18.0	75	20.8	24	18.6
Never smoked	2392	56.8	611	56.3	314	58.8	186	51.5	74	57.4
Functional index (Barthel)^e^										
Total or severe dependence	106	11.1	14	6.4	4	4.9	10	8.8	0	0
Moderate-mild dependence	294	30.9	63	28.6	29	35.4	21	18.4	11	64.7*
No dependence	552	58.0	143	65.0*	49	59.8	83	72.8*	6	35.3
Days from onset of symptoms to swabbing										
0–2 days	1569	37.2	465	42.8	261	48.9	128	35.5	47	36.2
3–4 days	1595	37.8	418	38.5	199	37.3	141	39.1	58	44.6
5–7 days	1053	25.0	203	18.7*	74	13.9*	92	25.5	25	19.2
Influenza vaccination ≥14 d from ILI onset	714	16.9	138	12.7*	46	8.6*	84	23.3*	5	3.8*

^a^ Comparison group, negative influenza admissions

^b^ Obesity was defined as a body mass index ≥30 for subjects ≥18 years of age and as a z-score of body mass index for age >2 standard deviations for subjects <18 years of age. Data were missing for 79 influenza-negative patients and 12 influenza-positive patients.

^c^ Data were missing for 4 influenza-negative patients and 1 influenza-positive patient.

^d^ Data were missing for 3 influenza negative patients and 1 influenza-positive patient.

^e^ Assessed for admissions ≥65 years of age. Data were missing for 21 influenza-negative patients and 12 influenza-positive patients.

*P<0.05 vs. influenza negative

In St. Petersburg and Moscow, A(H3N2) was the predominant circulating strain. A first wave due to A(H3N2) (68% of admissions in St. Petersburg and 66% in Moscow) with co-circulating A(H1N1)pdm09 (10% of admissions at both sites) was followed by a late wave of B/Yamagata lineage (7% in St. Petersburg and 19% in Moscow). Circulating strains in Turkey followed a similar pattern, with a first wave in which A(H3N2) predominated (80%) followed by a wave of B/Yamagata lineage (20%) but with no admissions due to A(H1N1)pdm09. In Beijing, influenza A(H3N2) (39%) co-circulated with influenza B/Yamagata lineage (43%) and A(H1N1)pdm09 (15%), and in Valencia, a first wave of A(H1N1)pdm09 (80% of admissions) was followed by a less intense late wave of A(H3N2) (18%) but with no admissions due to influenza B.

### Risk factors for admission with laboratory-confirmed influenza

The risk of hospital admission with influenza (vs. influenza-negative admission) was directly related to age, sex, and underlying medical conditions and inversely related to influenza vaccination and time elapsed from onset of symptoms to swabbing (Table 2, Table 3 and S4 Table).

**Table 3:** Risk of admission with influenza by site, strain, and patient-related characteristics.

		Influenza positive		Crude OR			Heterogeneity by strain (I^2^)	Adjusted OR		
Variable/characteristic	N	n	%	Value	95% CI			Value	95% CI	
Days from onset of symptoms to swabbing^a^										
0–2 days	2034	465	22.9	1.00			63.20%	1.00		
3–4 days	2013	418	20.8	0.88	0.76	1.03	72.10%	0.80	0.69	0.94
5–7 days	1256	203	16.2	0.65	0.54	0.78	19.40%	0.55	0.45	0.67
Age^b^										
0–5 months	557	54	9.7	1.00			0.0%	1.00		
6–11 months	315	36	11.4	1.20	0.77	1.88	0.0%	1.21	0.77	1.90
1–4 years	1086	181	16.7	1.86	1.35	2.57	0.0%	1.90	1.36	2.64
5–17 years	393	102	26.0	3.26	2.28	4.68	0.0%	3.64	2.50	5.30
18–49 years	1278	349	27.3	3.50	2.58	4.75	0.0%	3.95	2.84	5.49
50–64 years	469	132	28.1	3.65	2.58	5.15	68.6%	3.71	2.54	5.44
65–74 years	398	94	23.6	2.88	2.00	4.14	0.0%	3.13	2.07	4.74
75–84 years	532	106	19.9	2.32	1.63	3.30	0.0%	2.51	1.67	3.79
≥85 years	275	32	11.6	1.23	0.77	1.95	17.0%	1.39	0.83	2.32
Sex^c^										
Male	2927	539	18.4	1.00			0.0%	1.00		
Female	2376	547	23.0	1.33	1.16	1.51	0.0%	1.28	1.11	1.49
Female, non-pregnant	2039	391	19.1	1.05	0.91	1.21	0.0%	1.02	0.87	1.19
Other risk factors^d^										
One or more	1858	430	23.4	1.28	1.12	1.47	17.90%	1.29	1.05	1.58
Cardiovascular disease	874	189	21.62	1.37	1.14	1.65	39.6%	1.64	1.33	2.02
COPD	746	139	18.63	1.14	0.92	1.40	91.4%	1.31	1.03	1.67
Asthma	270	76	28.15	1.94	1.47	2.58	27.6%	2.25	1.67	3.03
Immunosuppression	54	16	29.63	2.09	1.15	3.77	48.4%	2.25	1.23	4.11
Diabetes	495	105	21.21	1.33	1.06	1.69	92.3%	1.69	1.29	2.23
Renal disease	224	57	25.45	1.69	1.23	2.32	21.4%	2.11	1.48	3.01
Rheumatologic disease	23	11	47.83	4.55	1.99	10.38	58.5%	5.55	2.34	13.16
Neuromuscular	113	19	16.81	1.00	0.61	1.66	28.5%	1.16	0.69	1.95
Malignancy (active)	159	29	18.24	1.11	0.73	1.67	65.3%	1.18	0.75	1.85
Liver disease	88	23	26.1	1.75	1.08	2.85	0.0%	1.94	1.18	3.19
Autoimmune disease	48	16	33.3	2.48	1.35	4.56	0.0%	2.97	1.58	5.59
Obese	496	123	24.8	1.64	1.31	2.05	81.8%	1.88	1.46	2.41
Pregnancy^e^	337	156	46.29	2.93	2.07	4.14	0.0%	3.84	2.48	5.94
With comorbidity	50	30	60.0	5.19	2.25	11.99	0.0%	11.90	2.29	61.70
No other conditions	287	126	43.9	2.64	1.80	3.88	0.0%	3.55	2.21	5.68
Influenza vaccination^f^	852	138	16.2	0.71	0.58	0.86	0.0%	0.61	0.48	0.77

Risk of admission with influenza was determined by comparing influenza-positive and influenza-negative admissions. Strains A(H3N2), A(H1N1)pdm09, and B/Yamagata lineage were considered. Admissions with not-subtyped strains or with B/Victoria lineage were not included because of small sample sizes. I^2^ for the aOR of influenza by site was 0.0%, and the clustering effect by site was P = 1.0^.^.

^a^ Adjusted by sex, age and calendar time splines, comorbidity, hospital admission in previous 12 months, vaccination during the current season, and time to swab.

^b^ Adjusted by health care access, influenza vaccination, underlying conditions, sex, and site as an indicator variable.

^c^ Adjusted by age, comorbidity, smoking habits, socioeconomic status, calendar time, and site as an indicator variable. For heterogeneity estimates, pregnant women were excluded from comparison groups.

^d^ For other risk factors, the first column indicates admissions with the risk factor and influenza positive and the second column indicates the number of subjects with the underlying condition included in the study. The comparison group to estimate the aOR of influenza admission for the risk factor (not shown) is included in admissions without underlying conditions or pregnant, with estimates adjusted for previous vaccination and hospitalizations during the previous 12 months.

^e^ Adjusted by social class, smoking habits, comorbidity, age, and calendar time.

^f^ Adjusted by age, sex, comorbidity, smoking habits, social class, calendar time, time to swab, and site as an indicator variable.

The risk of admission with influenza by site was similar as indicated by low heterogeneity (I^2^ = 0%) and a lack of a clustering effect (data not shown). Frequencies of influenza-positive results were 18.3% (230/1256) for St. Petersburg, 22.3% (281/1260) for Moscow, 23.5% (114/486) for Turkey, 21.4% (90/421) for Beijing, and 19.7% (371/1880) for Valencia.

### Age

The age distribution of admissions with confirmed influenza varied by site (S1 Fig), but overall, all age groups were represented in the pooled dataset (Table 2 and S1 Fig).

**Fig 2:**
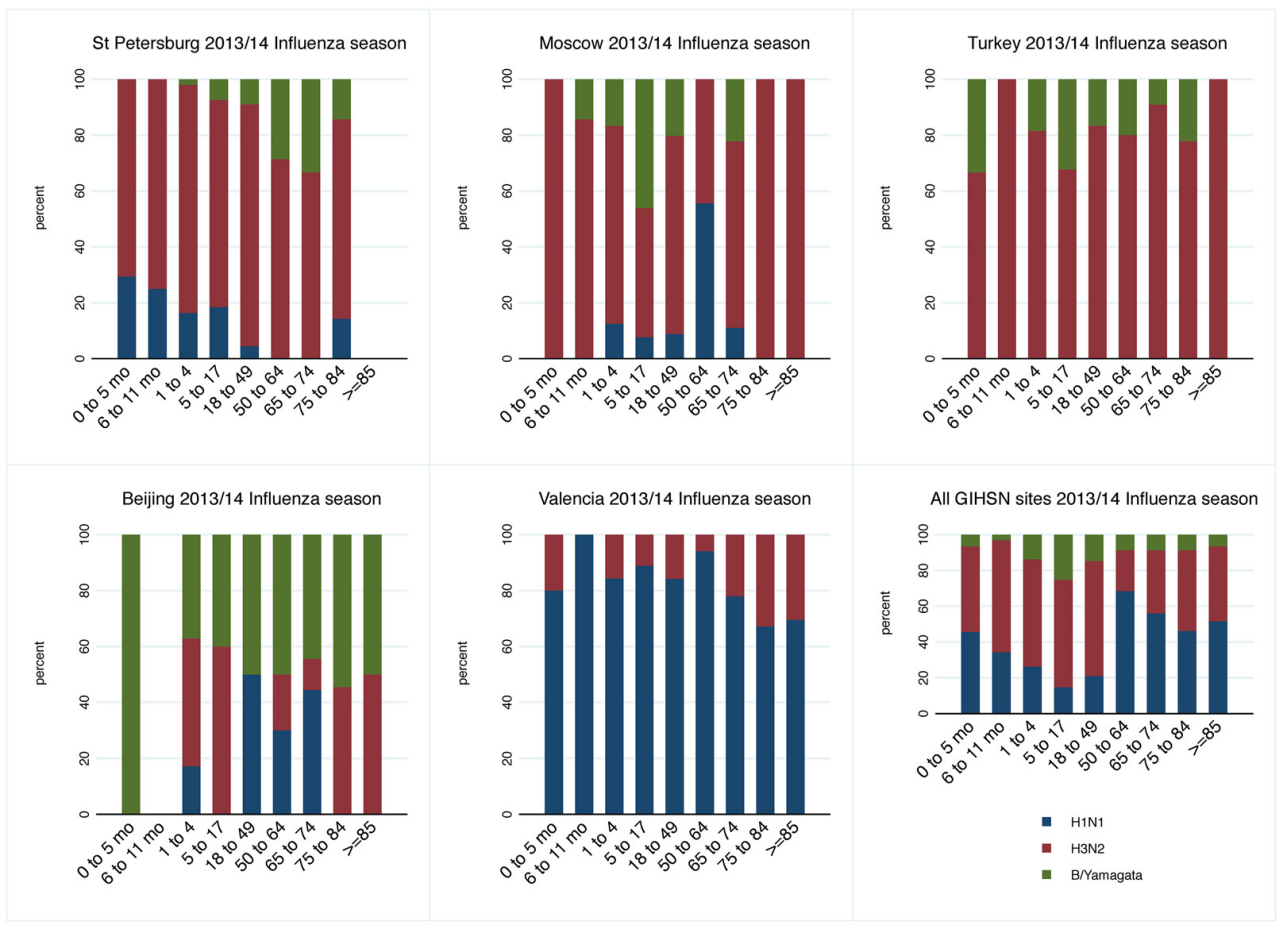
Distribution of positive admissions by site, strain, and age group.

The proportion of admissions with laboratory-confirmed influenza increased from 10% in patients <12 months of age to 28% in patients 50–64 years of age, whereas in the oldest age groups, influenza positivity decreased with age (24% in the 65–74 year age group to 12% in the >85 year age group; data not shown). Influenza A(H3N2), A(H1N1)pdm09, and B/Yamagata lineage were identified in patients of all ages (Fig 2). In St. Petersburg, Moscow, and Turkey, strain A(H3N2) dominated in all age groups except for the 50–64 year age group in Moscow. A(H1N1)pdm09 was predominant in patients <65 years of age, although in Valencia, it was the dominant strain in all groups. B/Yamagata lineage was largely detected in young patients in Moscow, Turkey, and Beijing. In Beijing, B/Yamagata lineage co-circulated with A(H3N2) and A(H1N1)pdm09 (Fig 1) but was the dominant strain overall, was observed in all age groups, and was the most common in patients ≥75 years of age (Fig 2).

Age was an independent risk factor for admission with influenza infection (Table 3). Significant heterogeneity by strain (I^2^ = 69%) was observed for the effects of age among the 50–64 year age group (Table 3 and S2 Fig), in which the probability of A(H1N1)pdm09 infection was higher than A(H3N2) or B/Yamagata lineage.

### Sex

We did not detect major differences in sex distribution by site (S4 Table; p = 0.1970) or by strain (Table 3, I^2^ = 0.0%). Overall, more influenza-positive admissions were female than male (23% vs. 18%; p<0.0001, Table 3). Excluding pregnant women, 19% of women and 18% of men were influenza positive. After adjustment, the risk of admission with influenza was similar in females and males (aOR = 1.02 [95% confidence interval (CI), 0.87–1.19]; I^2^ = 0.0%).

### Comorbidities

**Fig 3:**
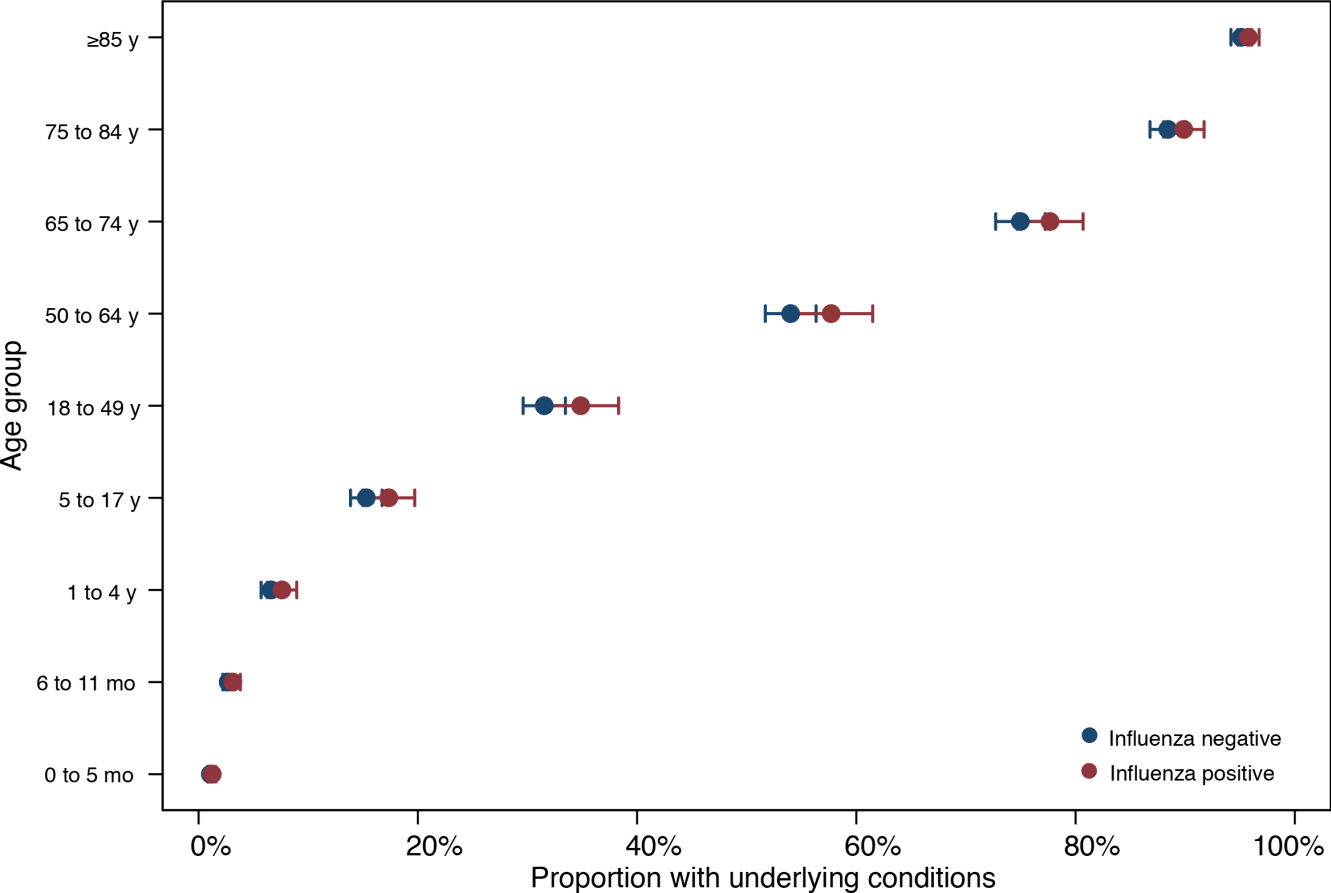
Proportion of admissions with underlying conditions by age group.

Of all admissions with influenza, 60% were in subjects without underlying chronic conditions (Table 2). The proportion of admissions with confirmed influenza without underlying conditions ranged from 96% for patients <1 year of age to 3% for patients ≥85 years of age (Fig 3). The remaining 40% of admissions with influenza were in patients with one or more underlying conditions. Patients with comorbidities were 30% more likely to be influenza-positive than patients without comorbidities (aOR = 1.29 [95% CI, 1.05–1.58]; Table 3). Overall, influenza was associated with a major risk of severe disease across age groups and underlying conditions (S2 Fig).

**Fig 4:**
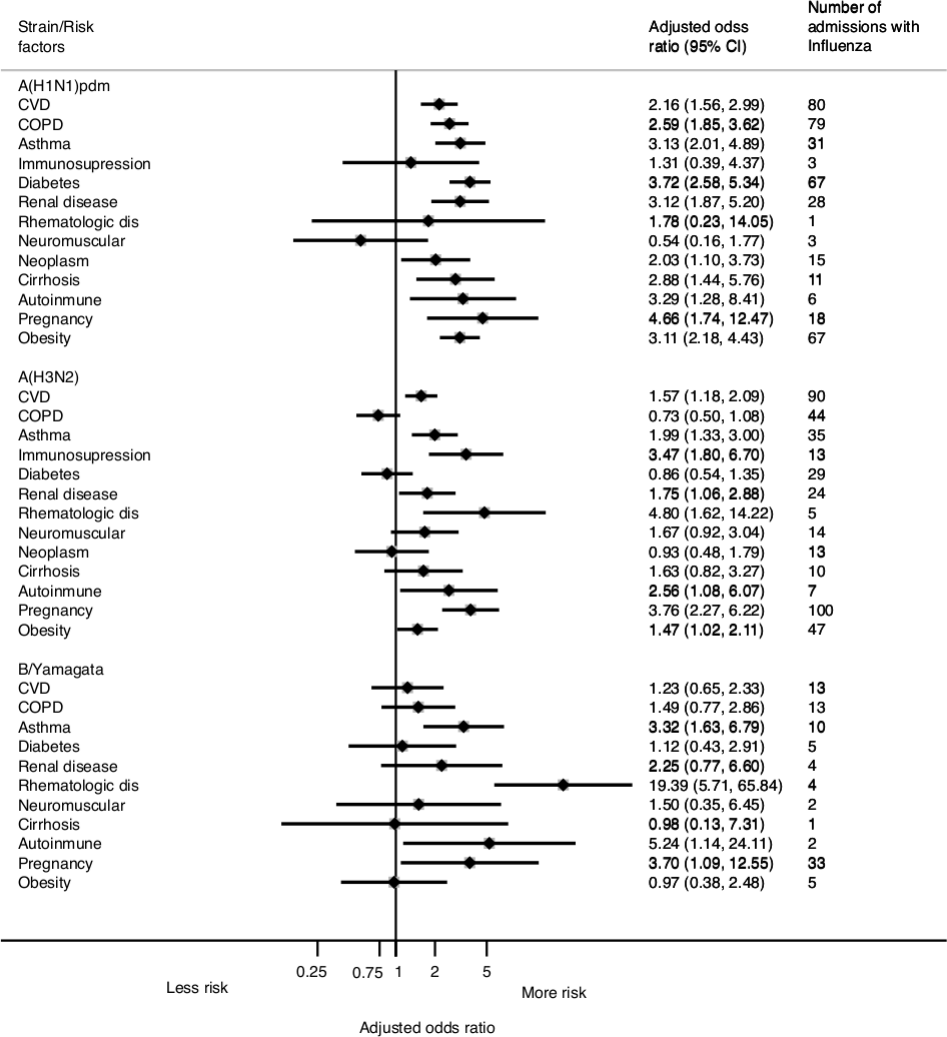
Risk of admission with influenza related to underlying conditions.

Compared with admissions in patients with no comorbidities, the risk (aOR) of severe influenza was increased in patients with cardiovascular disease (1.64 [95% CI, 1.33–2.02]), asthma (2.25 [95% CI, 1.67–3.03]), immunosuppression (2.25 [95% CI, 1.23–4.11]), renal disease (2.11 [95% CI, 1.48–3.01]), liver disease (1.94 [95% CI, 1.18–3.19]), autoimmune disease (2.97 [95% CI, 1.58–5.59]), or pregnancy (3.84 [95% CI, 2.48–5.94]). For most of comorbidities, the risk was similar for strains A(H3N2), A(H1N1), and B/Yamagata lineage (I^2^<50%; Table 3 and Fig 4), although the risk varied by strain (I^2^ >50%) and was mostly associated with A(H1N1)pdm09 for chronic obstructive pulmonary disease (COPD) (aOR = 2.59 [95% CI, 1.85–3.62]) and diabetes (aOR = 3.72 [95% CI, 2.58–5.34). In the case of obesity, the adjusted risk by strain was not homogenous (I^2^ = 82%) but was significant for A(H1N1)pdm09 (aOR = 3.11 [95% CI, 2.18–4.43]) and A(H3N2) (aOR = 1.47 [95% CI, 1.02–2.11). The number of B/Yamagata lineage admissions was similar for COPD, diabetes, and obesity, although few patients had these comorbidities (Fig 4).

### Pregnancy

A total of 627 admissions were women 15–45 years of age (393 in Moscow, 127 in St. Petersburg, and 82 in Valencia, 14 in Turkey, and 11 in Beijing). Of these, 327 in Moscow (83%), 1 in St. Petersburg (1%), and 8 in Valencia (11%) were pregnant. No pregnant women 15–45 years of age were recruited in Turkey or Beijing.

Pregnancy was a significant and independent risk factor for severe influenza disease. Amongst women 15–45 years of age, 46% of pregnant women vs. 23% of non-pregnant women were positive for influenza. In the subset of pregnant women with comorbidities, 60% of pregnant women vs. 22% of non-pregnant women were positive for influenza. The combination of pregnancy and comorbidity appeared to contribute to the development of severe influenza disease, although the interaction test was not significant (p = 0.0650). The aOR for admission with influenza in pregnant women vs. non-pregnant women was 11.90 (95% CI, 2.29–61.70) when comorbidities were present and 3.55 (95% CI, 2.21–5.68) in the absence of comorbidities (Table 3). This increased risk, independent of additional comorbidity, was observed for all three influenza viruses (I^2^ = 0.0%; Table 3 and Fig 4).

The rate of laboratory-confirmed influenza was 43% for pregnant women in the first trimester, 47% in the second trimester, and 47% in the third trimester. This pattern was similar across strains (p = 0.311) and for pregnancies with and without confirmed influenza (p = 0.859) (data not shown). Compared to non-pregnant women, the risk for hospitalization due to influenza (vs. influenza-negative hospitalization) was significantly higher during all trimesters of pregnancy. The aOR for influenza positivity was higher in the second (4.77 [95% CI, 2.40–9.53]) and third trimester (aOR = 4.72 [95% CI, 2.33–9.55]) than in the first trimester (4.18 [95% CI, 1.92–9.10]), although CIs were overlapping.

### Other risk factors, vaccination status

Of the 5303 patients included, 852 (16%) were vaccinated at least 14 days before symptoms onset. By site, the rates were 1% in St. Petersburg, 4% in Moscow, 11% in Turkey, 12% in Beijing, and 37% in Valencia (S4 Table). Overall, 138 (16.2%) admissions with influenza were in vaccinated subjects and 942 (21.4%) were in non-vaccinated subjects. The aOR of admission with influenza in vaccinated compared to unvaccinated subjects was 0.61 (95% CI [0.48–0.77]), with no heterogeneity by strain (I^2^ = 0.0%)

### Complications and outcomes in admissions with influenza

**Fig 5:**
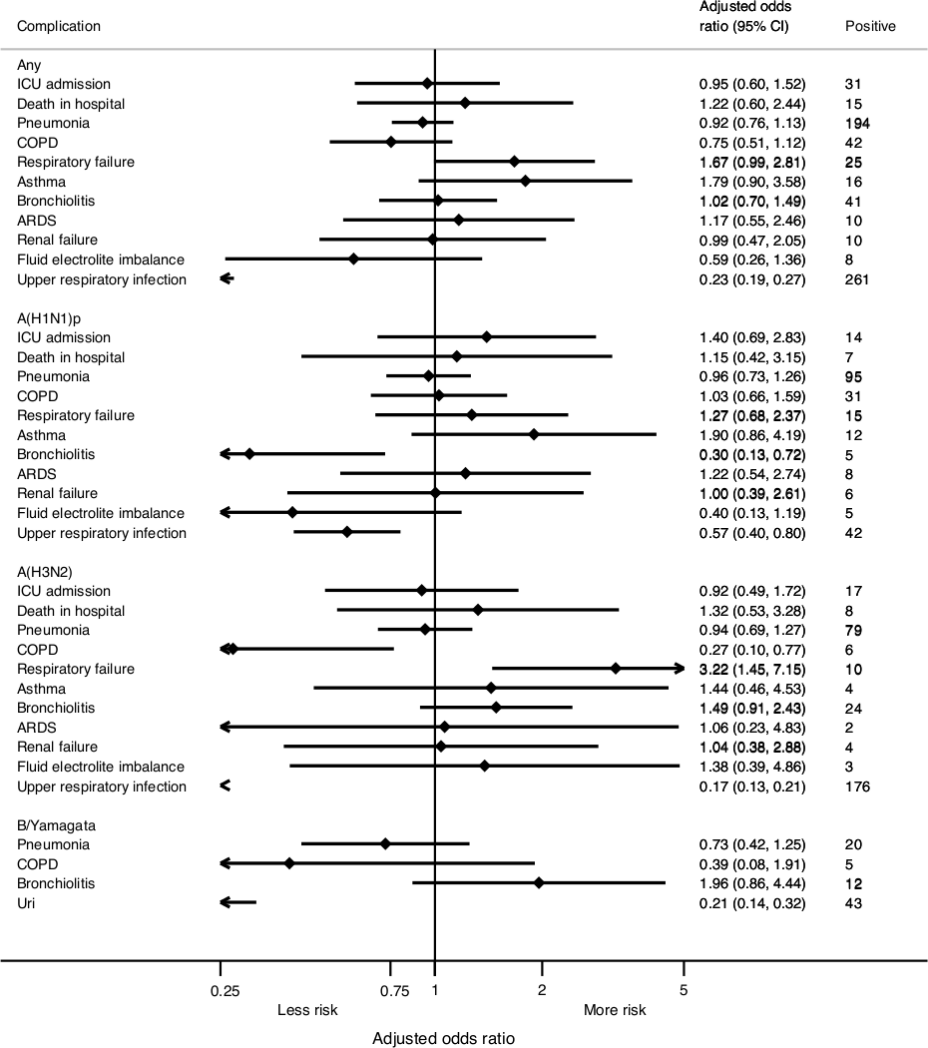
Outcomes and complications among admissions with influenza.

During the study period, 143 ICU admissions and 71 in-hospital deaths were recorded. Of the 143 ICU admissions, 31 (2.9%) were influenza-positive and 112 (2.7%) were influenza-negative (p = 0.8910). Of the 71 in-hospital deaths, 15 (1.4%) were influenza-positive and 56 (1.3%) were influenza-negative (p = 0.7220) (Table 4). Influenza infection was not a significant risk factor for ICU admission (aOR = 0.95 [95% CI, 0.60–1.52]) or death (aOR = 1.22 [0.60–2.44]) (Fig 5).

**Table 4:** Indicators of severity, complications, and major diagnoses at discharge.

	Negative		Influenza (all)		A(H1N1)pdm09		A(H3N2)^a^		B (all)^b^		
	N = 4217		N = 1086		N = 362		N = 574		N = 151		
Indicator	n	%	n	%	n	%	n	%	n	%	P-value
Indicator of severity											
ICU admission	112	2.7	31	2.9	14	3.9	17	3.0	0	0.0	0.7220
In-hospital death	56	1.3	15	1.4	7	1.9	8	1.4	0	0.0	0.8910
Length of stay, median (interquartile range)	6 (4–8)		6 (4–8)		5 (3–8)		6 (4–8)		6 (4–8)		0.2593
Pulmonary complications											0.0000
Pneumonia	744	17.6	194	17.9	95	26.3	79	13.8	20	13.2	
Exacerbation of COPD	183	4.3	42	3.9	31	8.6	6	1.0	5	3.3	
Respiratory failure	67	1.6	25	2.3	15	4.2	10	1.7	0	0.0	
Asthma exacerbation	32	0.8	16	1.5	12	3.3	4	0.7	0	0.0	
Pulmonary collapse	9	0.2	1	0.1	1	0.3	0	0.0	0	0.0	
Hemoptysis	1	0.0	0	0.0	0	0.0	0	0.0	0	0.0	
Acute respiratory distress syndrome	34	0.8	10	0.9	8	2.2	2	0.3	0	0.0	
Bronchiolitis	210	5.0	41	3.8	5	1.4	24	4.2	12	7.9	
Upper respiratory infection	1947	46.2	261	24.0	42	11.6	176	30.7	43	28.5	
Metabolic failure											0.1410
Acute renal failure	60	1.4	10	0.9	6	1.7	4	0.7	0	0.0	
Diabetic coma	5	0.1	0	0.0	0	0.0	0	0.0	0	0.0	
Fluid/electrolyte/acid-base/balance disorder	47	1.1	8	0.7	5	1.4	3	0.5	0	0.0	
Cardiovascular processes											0.0828
Acute myocardial infraction	4	0.1	0	0.0	0	0.0	0	0.0	0	0.0	
Acute heart failure	1	0.0	1	0.1	1	0.3	0	0.0	0	0.0	
Arterial or venous embolism	0	0.0	1	0.1	0	0.0	1	0.2	0	0.0	
Pulmonary embolism	1	0.0	0	0.0	0	0.0	0	0.0	0	0.0	
Cardiac arrest	0	0.0	1	0.1	0	0.0	1	0.2	0	0.0	
Malignant hypertension	16	0.4	5	0.5	3	0.8	2	0.3	0	0.0	
Systemic inflammatory response syndrome, shock, or disseminated intravascular coagulation	19	0.5	4	0.4	1	0.3	3	0.5	0	0.0	0.7080
Neurologic diagnoses											0.2430
Altered mental status	4	0.1	1	0.1	1	0.3	0	0.0	0	0.0	
Convulsions	1	0.0	0	0.0	0	0.0	0	0.0	0	0.0	
Meningitis	4	0.1	0	0.0	0	0.0	0	0.0	0	0.0	
Anoxic brain damage	0	0.0	1	0.1	0	0.0	1	0.2	0	0.0	
Major discharge diagnoses											
Influenza	114	2.7	487	44.8	161	44.6	269	46.9	57	37.7	0.0000
Pneumonia	801	19.0	99	9.1	34	9.4	52	9.1	13	8.6	
Other respiratory disease	2369	56.2	318	29.3	87	24.1	179	31.2	52	34.4	
Cardiovascular	231	5.5	48	4.4	27	7.5	20	3.5	1	0.7	
Other	702	16.6	134	12.3	52	14.4	54	9.4	28	18.5	

^a^ 40 non-subtyped A influenza were considered as A(H3N2)

^b^ 3 B/Victoria lineage and 18 non-subtyped B influenza viruses are grouped under B (all)

**Fig 6:**
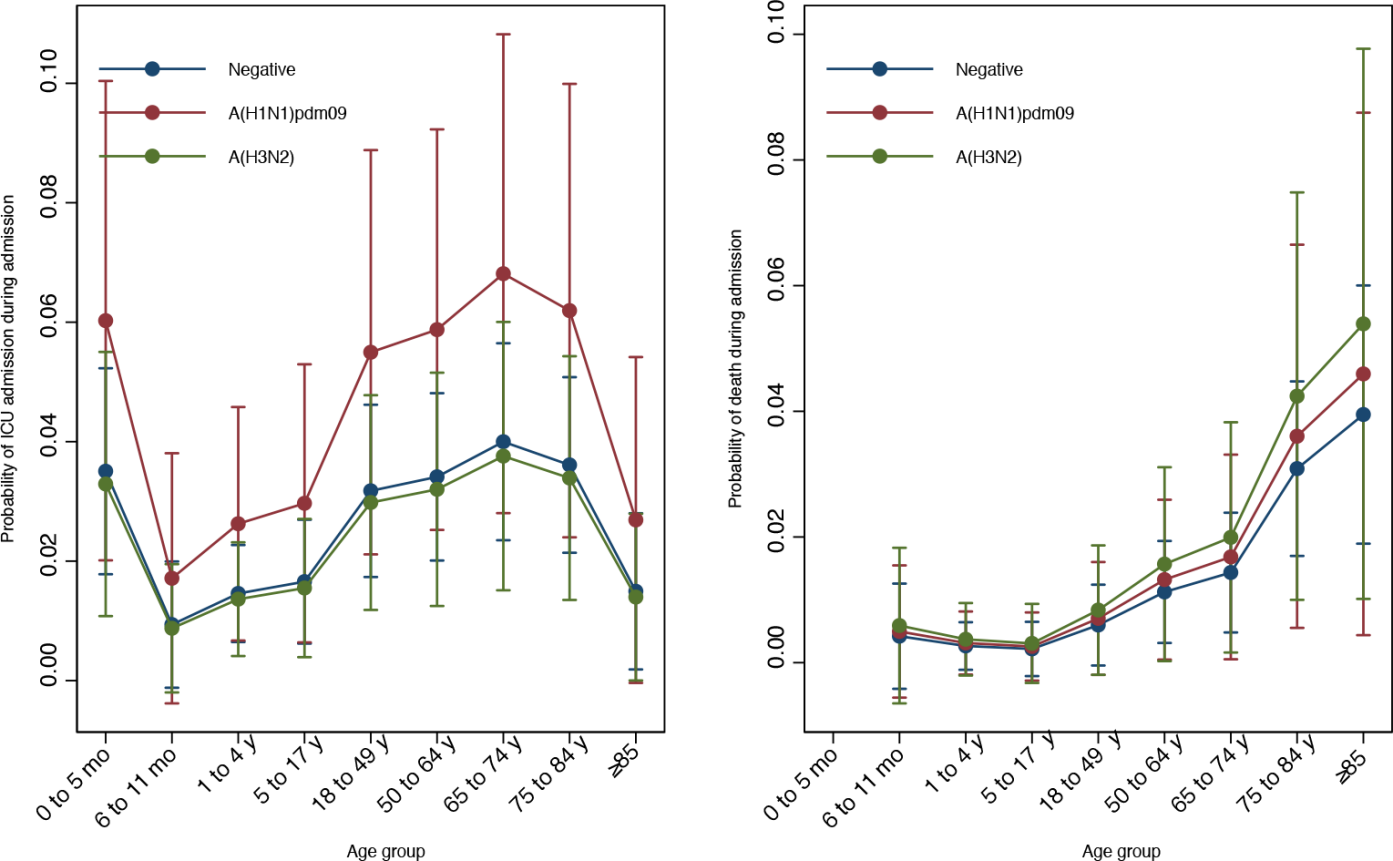
ICU admission and in hospital death by age group and RT-PCR result. Capped spikes show 95% confidence intervals.

ICU admission occurred in 14 (4%) patients infected with A(H1N1) and 17 (3%) patients infected with A(H3N2) compared to 112 (3%) influenza-negative patients. In-hospital deaths occurred in 7 (1.9%) patients infected with A(H1N1) and 8 (1.4%) patients infected with A(H3N2) compared to 56 (1.3%) influenza-negative patients (Table 4). Overall and by strain, there was a trend for influenza as a risk factor for ICU admission, mostly in patients infected with A(H1N1)pdm09, although 95% CIs were overlapping (Fig 6). We also observed a trend for death in patients infected with A(H3N2), although, again, 95% CIs were overlapping.

The mean length of hospital stay was similar between patients with and without confirmed influenza (Table 4) and between patients with A(H3N2), A(H1N1), or B/Yamagata lineage influenza infection (data not shown; p = 0.752).

At hospital discharge, 586 (54%) admissions with confirmed influenza were allocated discharge codes for influenza or pneumonia compared to 915 (22%) admissions without confirmed influenza (p<0.001) (Table 4). Another 182 (17%) admissions with confirmed influenza were allocated discharge codes unrelated to influenza or other respiratory conditions.

## Discussion

This study showed a prolonged and variable pattern of influenza circulation and severe disease during the 2013/2014 Northern hemisphere influenza season. The influenza strains varied between sites and, during the course of a full season, within each site. Over a 20-week span, influenza A was the predominant subtype, with more A(H3N2) than A(H1N1). Fewer hospitalizations were caused by B/Yamagata lineage, and the fewest were due to B/Victoria lineage. Three patterns of circulation were observed: A(H3N2)-dominant, A(H1N1)-dominant, and mixed. Most sites experienced more than one wave of influenza, with each wave due to different strains. Overall, these findings agree with the patterns of circulation reported by the World Health Organization for the 2013/2014 Northern hemisphere influenza season [28].

Hospital admissions with laboratory-confirmed influenza occurred in all age groups, although the probability of an influenza-positive result amongst admitted patients was lowest in the oldest and youngest age groups. We also observed a larger burden of non-influenza related hospitalizations in the youngest (< 1 year) and oldest (≥85 years) patients, which could explain the lower relative burden of influenza infection amongst all acute respiratory disease admissions in these age groups. This would also explain the age-dependent pattern of the odds ratio for influenza infection, which agrees with our results from 2012–2013 [6] and has also been reported in other studies [29,30] and in a recent extensive review [10]. In this study, we do not report age-specific influenza incidence rates, where a J- or U- shape curve would be expected [31,32] because data were not available on the catchment populations of study hospitals. Nevertheless, our results demonstrate that severe influenza is a significant threat for all age groups.

In this study, patients without underlying conditions accounted for 60% of admissions with influenza. These numbers are based on active surveillance using a common protocol, in contrast to surveillance based on the judgment of a clinician, where the percent of admissions without comorbidities can be much lower [33]. The fraction of influenza-positive patients without underlying conditions depended on age, with the highest rate (96%) in patients <1 year of age and the lowest rate (3%) in patients ≥85 years of age. A high rate of young children hospitalized with laboratory-confirmed influenza without comorbidities has been noted elsewhere [34,35]. Young adults without comorbidities were hospitalized with influenza at a high rate during the 2009 A(H1N1)pdm09 pandemic [36]. Thus, influenza is of broad public health relevance across all age groups and risk profiles.

Amongst the patients included in this study, having a comorbidity, especially asthma, increased the risk of being influenza positive by 30%, irrespective of influenza strain [37]. In some instances, such as in patients with COPD, diabetes, and obesity, this risk was specific for strain A(H1N1)pdm09. Our findings are consistent with the hypothesis that influenza causes severe disease by over-stimulating the immune response and exacerbating underlying conditions [38].

Pregnancy was a major and outstanding risk factor for influenza-positive admission, regardless of strain. Notably, the risk was elevated throughout all trimesters. Comorbidity increased the risk of influenza-positive admission in pregnant women several fold. These findings, based on a significant number of included admissions in pregnant women, strongly support the current recommendation that pregnant women be prioritized for influenza vaccination [39]. Our observations were the most recent of several demonstrating that pregnant women are at increased risk for severe seasonal influenza due to A(H1N1)pdm09 [40–44], and they provide information on the risk posed during pregnancy by A(H3N2) and B/Yamagata lineage infection not previously described elsewhere.

Our observations should help address some of the concerns about the quality of the evidence supporting the definition of groups at risk for severe influenza [10]. In addition, our observations showed that, after adjustment and regardless of strain, influenza vaccination was associated with a consistent reduction in the risk of hospitalization with influenza.

Finally, we did not find an association between confirmed influenza and ICU admission or in-hospital death. However, this was based on relatively few deaths and ICU admissions so that the analysis was underpowered.

### Considerations and limitations

Interpretation of these findings should be made in light of the fact that the number of patients was insufficient to perform calculations for certain risk factors or comorbidities or to analyze by certain subgroups, such as by age group. To perform these analyses with sufficient power and therefore draw meaningful conclusions, data will have to be pooled over several seasons. Such data pooling is only possible if a common core protocol is consistently applied over several seasons and across sites, as in the GIHSN. A pooled analysis awaits completion of data accrual and careful decisions about the best way to perform and present the results.

In this study, we did not perform follow-up after discharge as others have done [9]. We also did not have population denominators, so probabilities or risks were calculated in reference to included admissions and should not be interpreted as being necessarily representative of the full population for each country. Nonetheless, as with all analysis based on surveillance data, it is reasonable to make certain overall conclusions that apply to the population in general [45].

To limit false negatives, we excluded patients swabbed more than 7 days after symptom onset because we and others [46] have found an association between time after symptom onset and influenza positivity. This probably does not explain the proportion of influenza-negative patients and its variability with age. Furthermore, the adjusted probability of a positive result for influenza was homogeneous across sites. Although using a test-negative study to examine other risk factors for influenza might be problematic when the factors are also associated with the chance of inclusion in the control group, we used only admissions without co-morbidity as a comparison group, and our approach minimized selection bias and ensured comparability and exchangeability of comparison groups. Our approach should also have reduced information bias on outcome ascertainment and should substantially add to the precision of our estimates of odds ratios for hospitalization with influenza.

The protocol and study management used by GIHSN [14] is designed to address the majority of limitations identified for studies monitoring severe influenza [8–10]. In particular, we used an active and prospective ascertainment of eligible admissions, we followed a common core protocol, and we used the same inclusion criteria and case definition across sites. We also performed systematic sampling according to protocol instead of using clinician judgment, and we used RT-PCR to detect influenza viruses in combined nasopharyngeal swabs, two steps that have been described as adequate for surveillance schemes [47,48]. Accrual of information during consecutive seasons and growth of the GIHSN, coupled with application of this common core protocol, should allow us to pool data on strain effects across seasons and sites with minor site strain-subject effects and should allow for a more robust analysis of the severe consequences of influenza.

Hospital admission with influenza was used as a correlate of severe influenza to allow for non-selective objective and traceable enumeration. This also allowed for useful and relevant comparison groups for influenza (overall and by strain) for the existing risk group and for severe outcomes. Due to similarities in risk profiles between admissions with and without influenza [49], examining the distribution of risk factors among hospitalized subjects may not be ideal. However, admissions are considered a relevant outcome for public health policy purposes [11,12], and in the majority of cases, severe outcomes (ICU, prolonged stay, and death during hospitalization) are already included in the definition of severe influenza [9].

## Conclusions

The current analysis demonstrated that influenza causes severe outcomes during an extended period of the year in the Northern hemisphere, although the strains vary between sites and within sites over time. Our results confirmed the impact of previously identified risk groups for hospital admission with influenza. In particular, we provided needed data to better define the risk associated with pregnancy [10,41]. Our results also demonstrate that influenza infection substantially increases the risk of hospital admission throughout pregnancy, a risk that is further increased by underlying conditions.

The approach used by the GIHSN provides clear and relevant information on severe influenza epidemiology, by strain and different population groups. Progressive accrual of information by the GIHSN during ensuing seasons will provide a more precise and relevant definition of the burden of severe disease caused by influenza and will provide for a better understanding of influenza as an individual, local, and global health problem.

## Supporting Information

S1 Fig Age distribution by site and overall in the number of admissions by sex and by 5-year groups from 0 to 110 years of age. (TIF)

S2 Fig Adjusted risk of admission with influenza by age group and strain. (TIF)

S1 Table Characteristics of participating hospitals, 2013/2014 season. (DOCX)

S2 Table Presenting complaints used to identify admissions possibly related to an influenza infection. (DOCX)

S3 Table Protocol application across sites. (DOCX)

S4 Table Characteristics of included admissions by site and RT-PCR result. (DOCX)

S1 Text GIHSN laboratory characteristics and procedures. (DOCX)

## Abbreviations

CI: confidence interval
GIHSN: Global Influenza Hospital Surveillance Network
ICU: intensive care unit
ILI: influenza-like illness
RT-PCR: reverse transcription polymerase chain reaction
