# Supplementary figures and images for: Epidemiology of Hospital Admissions with Influenza during the 2013/2014 Northern Hemisphere Influenza Season: Results from the Global Influenza Hospital Surveillance Network
Source: PLoS One. 2016 May 19;11(5):e0154970. doi: 10.1371/journal.pone.0154970 (PMC4873033; doi:10.1371/journal.pone.0154970)

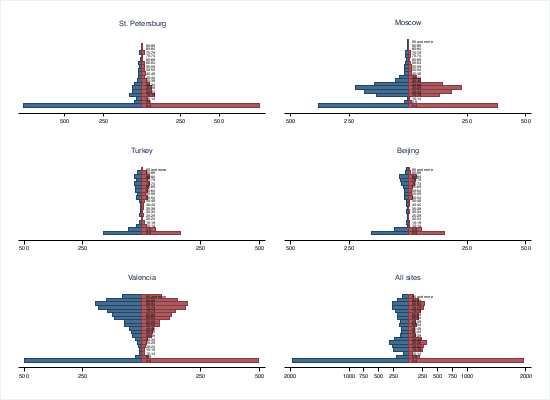

Supplement: S1 Fig — (TIF) [file pone.0154970.s001.tif]

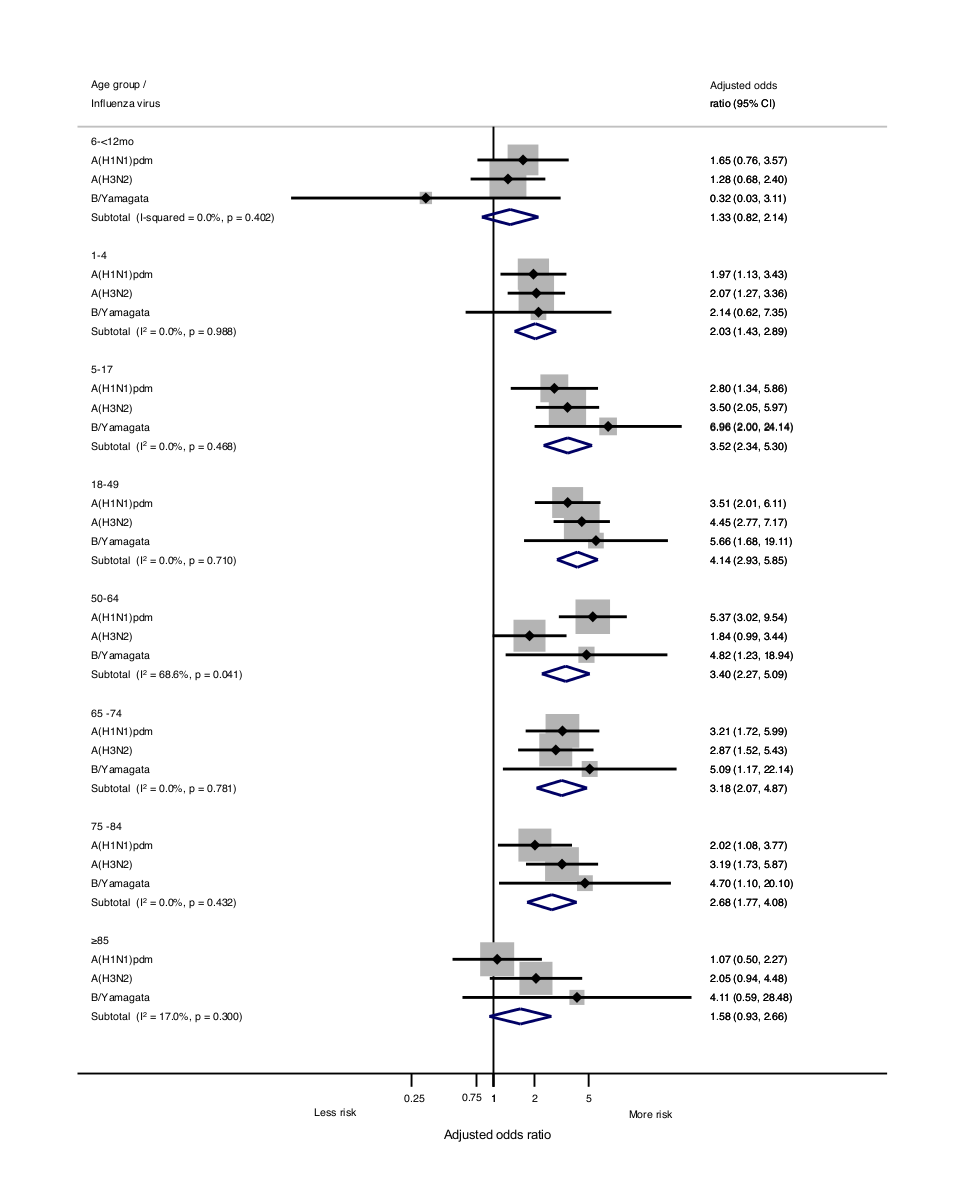

Supplement: S2 Fig — (TIF) [file pone.0154970.s002.tif]
