# Supplementary material for: Epidemiology of Hospital Admissions with Influenza during the 2013/2014 Northern Hemisphere Influenza Season: Results from the Global Influenza Hospital Surveillance Network
Source: PLoS One. 2016 May 19;11(5):e0154970. doi: 10.1371/journal.pone.0154970 (PMC4873033; doi:10.1371/journal.pone.0154970)
Supplement: S1 Table — (DOCX) [file pone.0154970.s003.docx]

**S1 Table. Characteristics of participating hospitals during the 2013/2014 season**

| Cities (Country) | Hospital | Type of hospital | Total no. of beds | No. of beds monitored during study^a^ | Type of wards monitored during study |
| --- | --- | --- | --- | --- | --- |
| Moscow (Russian Federation) | Emergency Respiratory Infection City Hospital #1 | Viral infectious disease | 860 | 297 | Acute respiratory disease, pediatric, pregnant, ICU |
| St Petersburg (Russian Federation) | City Infectious Diseases Hospital #30 | Infectious disease for adults | 300 | 60 | Adults (≥17 y) |
|  | Children’s Infectious Diseases Hospital #5 | Infectious disease for children | 650 | 120 | Infectious disease (0–16 y) |
|  | Children’s City Hospital #4 | General for children | 370 | 120 | Infectious disease (0–16 y) |
| Istanbul, Ankara, Bursa (Turkey | Hacettepe Univ. Adult Hospital | University | 561 | 50 | Acute medicine, emergency medicine |
|  | Gazi Univ. Hospital | University | 1068 | 47 | Adult emergency, infectious disease |
|  | Istanbul Faculty of Medicine | University | 1353 | 23 | Pediatrics: ICU, infectious, emergency, allergy |
|  | Istanbul Univ. Cerrahpaşa Hospital | University | 1656 | 38 | Pulmonology |
|  | Uludağ Univ. Pediatric Hospital | University pediatric | 108 | 69 | All medical wards, ICU |
|  | Dr. Siyami Ersek Hospital | Heart and vascular surgery  training and research | 480 | 174 | Cardiology |
|  | Dr. Lutfi Kirdar Kartal Research Hospital | Training and research | 750 | 336 | All medical wards, ICU |
| Beijng province (China) | Changping district hospital | General | 576 | 98 | Respiratory medicine, pediatrics, ICU |
|  | Beijing Huairou Hospital | General | 651 | 100 | Respiratory medicine, pediatrics |
| Valencia (Spain) | General de Castellón | General | 580 | 563 | All medical wards, ICU |
|  | La Plana | General | 251 | 128 | All medical wards, ICU |
|  | Doctor Pesset | General | 540 | 268 | All medical wards, ICU |
|  | San Juan | General | 350 | 280 | All medical wards, ICU |
|  | Elda | General | 514 | 453 | All medical wards, ICU |
|  | La Fe | General | 441 | 326 | All medical wards, ICU |

^a^ Medical beds excluding surgical beds
