# Supplementary material for: Epidemiology of Hospital Admissions with Influenza during the 2013/2014 Northern Hemisphere Influenza Season: Results from the Global Influenza Hospital Surveillance Network
Source: PLoS One. 2016 May 19;11(5):e0154970. doi: 10.1371/journal.pone.0154970 (PMC4873033; doi:10.1371/journal.pone.0154970)
Supplement: S2 Table — (DOCX) [file pone.0154970.s004.docx]

**S2 Table. Diagnoses and presenting complaints used to identify admissions possibly related with an influenza infection**

| Patients ≥5 years of age | ICD-9 Codes | ICD-10 Codes |
| --- | --- | --- |
| Acute respiratory infection | 382.9; 460–466 | J00–J06, J20–J22, H66.90 |
| Acute myocardial infarction or acute coronary syndrome | 410–411 and 413–414 | I20–I25.9 |
| Asthma | 493–493.92 | J45.2–J45.22, J45.9–J45.998, J44–J44.9 |
| Heart failure | 428–429.0 | I50–I50.9; I51.4 |
| Pneumonia and influenza | 480–488 | J09–J18 |
| Chronic obstructive pulmonary disease | 490, 491, 492, 496 | J40–J44.9 |
| Myalgia | 729.1 | M79.1 |
| Metabolic failure (diabetic coma, renal dysfunction, acid-base disturbances, alterations to the water balance) | 250.1–250.3; 584–586; 276–277 | E11.9, E10.9, E11.65, E10.65, E10.11, E11.01, E10.641, E11.641, E10.69, E11.00, E10.10, E11.69, N17.0, N17.1, N17.2, N17.8, N17.9, N18.1, N18.2, N18.3, N18.4, N18.5, N18.6 N18.9, N19, E87.0, E87.1, E87.2, E87.3, E87.4, E87.5, E87.6, E87.70, E87.71, E87.79, E86.0, E86.1 |
| Altered consciousness, convulsions, febrile convulsions | 780.01–780.02; 780.09; 780.31–780.32 | R40.20, R40.4, R40.0, R40.1, R56.00, R56.01 |
| Dyspnea/respiratory abnormality | 786.0 | R06.0, R06–R06.9 |
| Respiratory abnormality | 786.00 | R06.9 |
| Shortness of breath | 786.05 | R06.02 |
| Respiratory abnormality nec | 786.09 | R06.3, R06.00, R06.09, R06.83 |
| Respiratory symptoms/chest symptoms | 786.9 | R06.89 |
| Fever or fever unknown origin or non-specified | 780.6–780.60 | R50, R50.9 |
| Cough | 786.2 | R05 |
| Sepsis, Systemic inflammatory response syndrome | 995.90–995.94 | R65.10, R65.11, R65.20, A41.9 |
| Patients 0–4 years of age | ICD 9 Codes | ICD 10 Codes |
| Acute upper or lower respiratory disease | 382.9; 460–466 | J00–J06, J20–J22 |
| Dyspnea, breathing anomaly, shortness of breath, tachypnea | 786.0; 786.00; 786.05–786.07; 786.09; 786.9 | R06.0, R06, R06.9, R06.3, R06.00, R06.09, R06.83, R06.02, R06.82, R06.2, R06.89 |
| Asthma | 493–493.92 | J45.2–J45.22, J45.9–J45.998, J44–J44.9 |
| Pneumonia and influenza | 480 to 488 | J09–J18 |
| Heart failure | 428–429.0 | I50–I50.9; I51.4 |
| Myalgia | 729.1 | M79.1 |
| Altered consciousness, convulsions, febrile convulsions | 780.01–780.02; 780.09; 780.31–780.32 | R40.20, R40.4, R40.0, R40.1, R56.00, R56.01 |
| Fever or fever unknown origin or non-specified | 780.6–780.60 | R50, R50.9 |
| Cough | 786.2 | R05 |
| Gastrointestinal manifestations | 009.0; 009.3 | A09.0; A09.9 |
| Sepsis, systemic inflammatory response syndrome | 995.90–995.94 | R65.10, R65.11, R65.20, A41.9 |

Admissions possibly related to influenza infection were based on Hayden FG, de Jong MD: Human influenza: Pathogenesis, clinical features, and management; In Webster RG, Monto AS, Braciale TJ, Lamb RA, editors. Textbook of Influenza. Chichester, West Sussex, UK, Wiley Blackwell, 2013; 374–391.
