# Supplementary material for: Epidemiology of Hospital Admissions with Influenza during the 2013/2014 Northern Hemisphere Influenza Season: Results from the Global Influenza Hospital Surveillance Network
Source: PLoS One. 2016 May 19;11(5):e0154970. doi: 10.1371/journal.pone.0154970 (PMC4873033; doi:10.1371/journal.pone.0154970)
Supplement: S3 Table — (DOCX) [file pone.0154970.s005.docx]

**S3 Table. Protocol application across sites**

| **Characteristic** | **Valencia (Spain)** | **St. Petersburg (Russian Federation)** | **Moscow (Russian Federation)** | **Istanbul, Ankara, Bursa (Turkey)** | **Beijing (China)** |
| --- | --- | --- | --- | --- | --- |
|  |  |  |  |  |  |
| Screening diagnosis | Admission diagnosis possibly associated with an influenza infection (Table S2) | Admission diagnosis: Influenza, acute respiratory illiness, severe acute respiratory infections, influenza-like illness, pneumonia, croup, and bronchitis | Admission diagnosis possibly associated with an influenza infection (Table S2) | Admission diagnosis possibly associated with an influenza infection (Table S2) | Admission diagnosis possibly associated with an influenza infection (Table S2) |
|  |  |  |  |  |  |
| Residency definition | Pertaining to hospital catchment area and resident for at least 6 months | Resident of St. Petersburg ≥ 6 months | Resident of Moscow ≥ 6 months, and has not been away for more 1 month | Resident of the city where the hospital is located≥ 6 months | Resident of the district of where the hospital is located ≥6 months |
|  |  |  |  |  |  |
| Recruited by | Full-time trained nurses | Doctors | Doctors, nurses, head of wards | Doctors, resident doctors | Doctors, nurses |
|  |  |  |  |  |  |
| Study start criteria | 2 consecutive weeks with ≥2 cases | Week with ≥5 laboratory-confirmed influenza cases | 2 consecutive weeks with ≥1 cases | 2 consecutive weeks with ≥1 cases | Defined by the national surveillance system |
|  |  |  |  |  |  |
| Study end criteria | 2 consecutive weeks with <3 cases | Week with no laboratory-confirmed influenza cases | Week with no laboratory-confirmed influenza cases | 2 consecutive weeks with no cases | Defined by the national surveillance system |
|  |  |  |  |  |  |
| Actual study period (epidemiological weeks with flu cases ascertained) | 2013 wk 51 –  2014 wk 11 | 2014 wk 04 –  2014 wk 20 | 2014 wk 2 –  2014 wk 23 | 2013 wk 49 –  2014 wk 14 | 2014 wk 02 –  2014 wk 16 |
|  |  |  |  |  |  |
