# Supplementary material for: Epidemiology of Hospital Admissions with Influenza during the 2013/2014 Northern Hemisphere Influenza Season: Results from the Global Influenza Hospital Surveillance Network
Source: PLoS One. 2016 May 19;11(5):e0154970. doi: 10.1371/journal.pone.0154970 (PMC4873033; doi:10.1371/journal.pone.0154970)
Supplement: S4 Table — (DOCX) [file pone.0154970.s006.docx]

**S4 Table. Characteristics of included admissions by site and influenza RT-PCR result**

| **Site** | **St Petersburg** | | | | **Moscow** | | | | **Turkey** | | | | **China** | | | | **Valencia** | | | |
| --- | --- | --- | --- | --- | --- | --- | --- | --- | --- | --- | --- | --- | --- | --- | --- | --- | --- | --- | --- | --- |
| **RT-PCR result for influenza** | **Negative** | | **Positive** | | **Negative** | | **Positive** | | **Negative** | | **Positive** | | **Negative** | | **Positive** | | **Negative** | | **Positive** | |
|  | **1026** | | **230** | | **979** | | **281** | | **372** | | **114** | | **331** | | **90** | | **1509** | | **371** | |
|  | **N** | **%** | **N** | **%** | **N** | **%** | **N** | **%** | **N** | **%** | **N** | **%** | **N** | **%** | **N** | **%** | **N** | **%** | **N** | **%** |
| Age (years) |  |  |  |  |  |  |  |  |  |  |  |  |  |  |  |  |  |  |  |  |
| Mean (standard deviation) | 11.7 | (17.9) | **18.8*** | (21.0) | 19.4 | (16.2) | **28.8*** | (17.7) | 36.1 | (33.4) | **27.2*** | (30.4) | 34.8 | (33.7) | 32.4 | (33.7) | 49.4 | (34.9) | 52.5 | (28.5) |
| Age group (years)***** |  |  |  |  |  |  |  |  |  |  |  |  |  |  |  |  |  |  |  |  |
| 0 to 5 mo | 213 | 20.8 | 25 | 10.9 | 44 | 4.5 | 2 | 0.7 | 41 | 11.0 | 6 | 5.3 | 4 | 1.2 | 1 | 1.10 | 201 | 13.3 | 20 | 5.4 |
| 6 to 11 mo | 109 | 10.6 | 15 | 6.5 | 65 | 6.6 | 8 | 2.8 | 30 | 8.1 | 5 | 4.4 | 18 | 5.4 | 0 | 0.00 | 57 | 3.8 | 8 | 2.2 |
| 1 to 4 | 343 | 33.4 | 59 | 25.7 | 233 | 23.8 | 28 | 10.0 | 53 | 14.2 | 27 | 23.7 | 98 | 29.6 | 35 | 38.90 | 178 | 11.8 | 32 | 8.6 |
| 5 to 17 | 80 | 7.8 | 32 | 13.9 | 71 | 7.3 | 15 | 5.3 | 57 | 15.3 | 31 | 27.2 | 49 | 14.8 | 15 | 16.70 | 34 | 2.3 | 9 | 2.4 |
| 18 to 49 | 222 | 21.6 | 73 | 31.7 | 521 | 53.2 | 200 | 71.2 | 25 | 6.7 | 12 | 10.5 | 26 | 7.9 | 4 | 4.40 | 135 | 8.9 | 60 | 16.2 |
| 50 to 64 | 36 | 3.5 | 16 | 7.0 | 32 | 3.3 | 10 | 3.6 | 56 | 15.1 | 10 | 8.8 | 34 | 10.3 | 10 | 11.10 | 179 | 11.9 | 86 | 23.2 |
| 65 to 74 | 8 | 0.8 | 3 | 1.3 | 7 | 0.7 | 9 | 3.2 | 46 | 12.4 | 11 | 9.6 | 35 | 10.6 | 10 | 11.10 | 208 | 13.8 | 61 | 16.4 |
| 75 to 84 | 14 | 1.4 | 7 | 3.0 | 4 | 0.4 | 8 | 2.8 | 46 | 12.4 | 9 | 7.9 | 55 | 16.6 | 11 | 12.20 | 307 | 20.3 | 71 | 19.1 |
| >=85 | 1 | 0.1 | 0 | 0.0 | 2 | 0.2 | 1 | 0.4 | 18 | 4.8 | 3 | 2.6 | 12 | 3.6 | 4 | 4.40 | 210 | 13.9 | 24 | 6.5 |
| Female | 441 | 43.0 | 104 | 45.2 | 402 | 41.1 | 191 | **68.0*** | 164 | 44.1 | 53 | 46.5 | 139 | 42 | 34 | 37.8 | 683 | 45.3 | 165 | 44.5 |
| Comorbidities (n) |  |  |  |  |  |  |  |  |  |  |  |  |  |  |  |  |  |  |  |  |
| None | 931 | 90.7 | 204 | 88.7 | 883 | 90.2 | 218 | 77.6 | 125 | 33.6 | 35 | 30.7 | 216 | 65.3 | 61 | 67.8 | 634 | 42 | 138 | 37.2 |
| One | 70 | 6.8 | 15 | 6.5 | 80 | 8.2 | 51 | **18.1*** | 127 | 34.1 | 48 | 42.1 | 60 | 18.1 | 17 | 18.9 | 395 | 26.2 | 129 | **34.8*** |
| More than one | 25 | 2.4 | 11 | 4.8 | 16 | 1.6 | 12 | 4.3 | 120 | 32.3 | 31 | 27.2 | 55 | 16.6 | 12 | 13.3 | 480 | 31.8 | 104 | 28 |
| Chronic diseases |  |  |  |  |  |  |  |  |  |  |  |  |  |  |  |  |  |  |  |  |
| Cardiovascular | 46 | 4.5 | 16 | 7 | 42 | 4.3 | 28 | **10.0*** | 111 | 29.8 | 29 | 25.4 | 81 | 24.5 | 16 | 17.8 | 405 | 26.8 | 100 | 27 |
| COPD | 10 | 1.0 | 7 | **3.0*** | 7 | 0.7 | 8 | **2.8*** | 89 | 23.9 | 14 | **12.3*** | 68 | 20.5 | 17 | 18.9 | 433 | 28.7 | 93 | 25.1 |
| Asthma | 16 | 1.6 | 4 | 1.7 | 17 | 1.7 | 5 | 1.8 | 67 | 18.0 | 30 | 26.3 | 3 | 0.9 | 2 | 2.2 | 91 | 6.0 | 35 | **9.4*** |
| Immunodeficiency | 7 | 0.7 | 2 | 0.9 | 0 |  | 0 |  | 11 | 3.0 | 9 | **7.9*** | 1 | 0.3 | 0 | 0 | 19 | 1.3 | 5 | 1.3 |
| Diabetes | 8 | 0.8 | 4 | 1.7 | 7 | 0.7 | 4 | 1.4 | 48 | 12.9 | 11 | 9.6 | 17 | 5.1 | 2 | 2.2 | 310 | 20.5 | 84 | 22.6 |
| Renal impairment | 5 | 0.5 | 2 | 0.9 | 14 | 1.4 | 12 | **4.3*** | 21 | 5.6 | 8 | 7.0 | 6 | 1.8 | 2 | 2.2 | 121 | 8 | 33 | 8.9 |
| Rheumatologic disease | 2 | 0.2 | 3 | **1.3*** | 1 | 0.1 | 2 | 0.7 | 9 | 2.4 | 5 | 4.4 | 0 | 0 | 1 | 1.1 | 0 |  | 0 |  |
| Neuromuscular | 13 | 1.3 | 1 | 0.4 | 4 | 0.4 | 1 | 0.4 | 29 | 7.8 | 9 | 7.9 | 1 | 0.3 | 1 | 1.1 | 47 | 3.1 | 7 | 1.9 |
| Active neoplasm | 3 | 0.3 | 2 | 0.9 | 10 | 1.0 | 2 | 0.7 | 34 | 9.1 | 7 | 6.1 | 1 | 0.3 | 1 | 1.1 | 82 | 5.4 | 17 | 4.6 |
| Liver disease | 11 | 1.1 | 2 | 0.9 | 10 | 1.0 | 9 | **3.2*** | 7 | 1.9 | 2 | 1.8 | 1 | 0.3 | 1 | 1.1 | 36 | 2.4 | 9 | 2.4 |
| Autoimmune | 0 | 0.0 | 3 | **1.3*** | 2 | 0.2 | 7 | **2.5*** | 4 | 1.1 | 0 | 0 | 1 | 0.3 | 1 | 1.1 | 25 | 1.7 | 5 | 1.3 |
| Pregnant^a^ | 0 | 0.0 | 1 | 3.4 | 179 | 77.2 | 148 | **91.9*** | 0 |  | 0 |  | 0 |  | 0 |  | 2 | 3.6 | 7 | **25.9*** |
| Obesity^b^ | 81 | 7.9 | 17 | 7.4 | 183 | 19.3 | 45 | 16.3 | 56 | 15.9 | 12 | 10.8 | 27 | 8.5 | 15 | 17.2 | 354 | 23.6 | 105 | 28.4 |
| Outpatient visits last 3 months |  |  |  |  |  |  |  |  |  |  |  |  |  |  |  |  |  |  |  |  |
| None | 585 | 57.1 | 139 | 60.7 | 364 | 37.2 | 68 | 24.2 | 61 | 16.4 | 23 | 20.2 | 1 | 0.3 | 0 | 0 | 272 | 18 | 76 | 20.5 |
| One | 229 | 22.4 | 41 | 17.9 | 137 | 14 | 27 | 9.6 | 61 | 16.4 | 19 | 16.7 | 135 | 41 | 34 | 37.8 | 357 | 23.7 | 120 | 32.3 |
| More than one | 210 | 20.5 | 49 | 21.4 | 478 | 48.8 | 186 | **66.2*** | 250 | 67.2 | 72 | 63.2 | 193 | 58.7 | 56 | 62.2 | 880 | 58.3 | 175 | 47.2 |
| Smoking habits |  |  |  |  |  |  |  |  |  |  |  |  |  |  |  |  |  |  |  |  |
| Current smoker | 249 | 24.3 | 55 | 23.9 | 379 | 38.8 | 59 | 21.0 | 76 | 20.4 | 33 | 28.9 | 86 | 26.1 | 26 | 29.2 | 219 | 14.5 | 101 | 27.2 |
| Past smoker | 168 | 16.4 | 37 | 16.1 | 116 | 11.9 | 50 | **17.8*** | 103 | 27.7 | 16 | **14.0*** | 54 | 16.4 | 14 | 15.7 | 372 | 24.7 | 83 | 22.4 |
| Never smoker | 609 | 59.4 | 138 | 60 | 483 | 49.4 | 172 | **61.2*** | 193 | 51.9 | 65 | 57.0 | 189 | 57.4 | 49 | 55.1 | 918 | 60.8 | 187 | **50.4*** |
| Dependence^c^ |  |  |  |  |  |  |  |  |  |  |  |  |  |  |  |  |  |  |  |  |
| Total or severe | 0 |  | 0 |  | 0 |  | 0 |  | 17 | 16.5 | 2 | 10.0 | 11 | 11.2 | 0 | 0 | 78 | 10.8 | 12 | 7.7 |
| Moderate-mild | 7 | 43.8 | 4 | 66.7 | 5 | 50.0 | 3 | 20.0 | 30 | 29.1 | 4 | 20.0 | 76 | 77.6 | 19 | 82.6 | 176 | 24.3 | 33 | 21.2 |
| None | 9 | 56.2 | 2 | 33.3 | 5 | 50.0 | 12 | 80.0 | 56 | 54.4 | 14 | 70.0 | 11 | 11.2 | 4 | 17.4 | 471 | 65 | 111 | 71.2 |
| Days to swabbing |  |  |  |  |  |  |  |  |  |  |  |  |  |  |  |  |  |  |  |  |
| 0-2 days | 613 | 59.7 | 122 | 53 | 431 | 44 | 163 | 58 | 77 | 20.7 | 31 | 27.2 | 102 | 30.8 | 30 | 33.3 | 346 | 22.9 | 119 | 32.1 |
| 3-4 days | 278 | 27.1 | 79 | **34.3*** | 400 | 40.9 | 99 | 35.2 | 137 | 36.8 | 50 | 43.9 | 109 | 32.9 | 30 | 33.3 | 671 | 44.5 | 160 | 43.1 |
| 5-7 days | 135 | 13.2 | 29 | 12.6 | 148 | 15.1 | 19 | **6.8*** | 158 | 42.5 | 33 | **28.9*** | 120 | 36.3 | 30 | 33.3 | 492 | 32.6 | 92 | **24.8*** |
| Influenza vaccination | 6 | 0.6 | 1 | 0.4 | 48 | 4.9 | 6 | **2.1*** | 41 | 11.0 | 7 | 6.1 | 44 | 13.3 | 7 | 7.8 | 575 | 38.1 | 117 | **31.5*** |

^a^ Admissions in women 15 to 45 years of age.

^b^ For patients ≥18 years of age, obesity was defined as body mass index ≥30; for patients <18 years age, obesity was defined as a z-score for body mass index for age > two standard deviations.

^c^ Admissions ≥65 years of age
