# Supplementary material for: Epidemiology of Hospital Admissions with Influenza during the 2013/2014 Northern Hemisphere Influenza Season: Results from the Global Influenza Hospital Surveillance Network
Source: PLoS One. 2016 May 19;11(5):e0154970. doi: 10.1371/journal.pone.0154970 (PMC4873033; doi:10.1371/journal.pone.0154970)
Supplement: S1 Text — (DOCX) [file pone.0154970.s007.docx]

# Supporting Methods

### GIHSN laboratory characteristics and procedures

The following laboratories analyzed the samples: Molecular Virology Laboratory of FISABIO, Valencia (Spain); WHO National Influenza Centre, D.I. Ivanovsky Research Institute of Virology of the Ministry of Health of the Russian Federation, Moscow (Russian Federation); WHO National Influenza Centre, Research Institute of Influenza from the Ministry of Health, St. Petersburg (Russian Federation); and the National Influenza Reference Laboratory, Istanbul Faculty of Medicine (Turkey). All of these are National Influenza Centers except for the Molecular Virology Laboratory of FISABIO in Valencia [1]. The coordinating site in China analyzed the samples from the Changping District hospital in the laboratory of Changping District Center for Disease Control and Prevention (CDC) in Beijing, the samples from the Beijing Huairou Hospital, the samples from the First People’s Hospital of Huzhou in the laboratory of Huzhou Prefecture CDC, and the samples from Ningbo Women and Children’s Hospital and Jiangshan Hospital in the laboratory of Ningbo Municipal CDC. All these laboratories belong to the National Influenza Surveillance Laboratory Network in China.

### Laboratories in the National Influenza Surveillance Laboratory Network, Beijing, China

Two pharyngeal swabs were obtained from each included patient using flocked swabs (Yocon, China). Samples were introduced into vials with 3 mL universal viral transport medium (UTM, Yocon) and kept at –20ºC until shipped to the laboratory. RNA extraction was performed from 140 μL sampling solution using QIAamp Viral RNA Mini Kit (Qiagen, Denmark) according to the manufacturer's instructions. RNA was eluted with 50 μL RNase-free water.

For influenza A and B detection, primers were designed basing on the sequence supplied by CNIC for the matrix protein (see supplemental data, Table 1). The tests were performed by rRT-PCR using an AgPath-ID One-Step RT-PCR kit (Applied Biosystems, USA) and a 7500 Fast Real-Time PCR System (Applied Biosystems) using 5 μL of RNA according to the manufacturer’s instructions and the WHO protocol [1]. Human ribonucleoprotein P gene was included as an internal control for the quality control of the sample, extraction, and amplification. Samples with a Ct value < 35 were considered as positive and hose with a Ct value > 37 were considered as negative. In all other cases, samples were retested to obtain a positive or negative result. Laboratory procedures to prevent PCR contamination were strictly followed and positive controls (purified viral nucleic acids from virus-infected cells) and negative controls (without sample and/or nucleic acid) were included.

For influenza A-positive samples, a typing rRT-PCR assay was performed as described above. The hemagglutinin (HA) gene with different probes (influenza A H1N1pdm09, H3, H5 and H7N9, listed in Table 1) was used for virus typing. For influenza B-positive samples, rRT-PCR was performed for the HA gene to distinguish B/Yamagata and B/Victoria lineages (Table 1).

Isolation of influenza viruses was carried out on Madin-Darby canine kidney cell culture. All isolates were typed and subtyped by hemagglutination and hemaggutination inhibition (HI) tests using guinea-pig red blood cells as described previously [2]. All strains with a hemaggutination inhibition titer > 8 were stored.

**Primers and probes for real-time RT-PCR**

| **Target** | **Forward primer sequence**  **(5’ to 3’)** | **Reverse primer sequence**  **(5’ to 3’)** | **Probe sequence (5’ to 3’)** |
| --- | --- | --- | --- |
| M gene of influenza A virus | GACCRATCCTGTCACCTCTGAC | GGGCATTYTGGACAAAKCGTCTACG | FAM-TGCAGTCCTCGCTCACTGGGCACG-BHQ1 |
| M gene of influenza B virus | TCCTCAACTCACTCTTCGAGCG | CGGTGCTCTTGACCAAATTGG | FAM-CCAATTCGAGCAGCTGAAACTGCGGTG-BHQ1 |
| HA gene of influenza A(H1N1 09pdm) virus | GACAAAATAACAAACGAAGCAACTGG | GGGAGGCTGGTGTTTATAGCACC | FAM-GCATTCGCAATGGAAAGAAATGCTGG-BHQ1 |
| HA gene of influenza A(H3) virus | ACCCTCAGTGTGATGGCTTCCAAA | TAAGGGAGGCATAATCCGGCACAT | FAM-ACGCAGCAAAGCCTACAGCAACTGT-BHQ1 |
| HA gene of influenza A(H5) virus | TGGAAAGTRTAARAAACGGAACGT | YGCTAGGGARCTCGCCACTG | FAM-TACCCGCAGTATTCAGAAGAAGC-BHQ1 |
| HA gene of influenza A(H7N9) virus | AGAAATGAAATGGCTCCTGTCAA | GGTTTTTTCTTGTATTTTTATATGACTTAG | FAM-AGATAATGCTGCATTCCCGCAGATG-BHQ1 |
| HA gene of influenza B(VIC) virus | AGACCAGAGGGAAACTATGCCC | TCCGGATGTAACAGGTCTGACTT | FAM-CAGACCAAAATGCACGGGGAAHATACC-BHQ1 |
| HA gene of influenza B(YAM) virus | AGACCAGAGGGAAACTATGCCC | TCCGGATGTAACAGGTCTGACTT | FAM-CAGRCCAATGTGTGTGGGGAYCACACC-BHQ1 |

### Molecular Virology Laboratory of FISABIO, Valencia, Spain

A nasopharyngeal and a pharyngeal swab were obtained from each included patient using flocked swabs (Copan, Italy). Samples were introduced into vials with 3 mL universal viral transport medium (UTM, Copan) and kept at –20ºC until shipped to the reference laboratory.

Total nucleic acids were extracted from one-third (1 mL) of the sample volume using an automated silica-based method (Nuclisens Easy-Mag, bioMérieux, Lyon, France) and the generic 2.0.1 protocol. The elution volume was 50 μL.

Four different multiplex real-time RT-PCR screening assays were used as described below. Reactions were performed in a Lightcycler 480II apparatus (Roche) using 5 mL of the eluted nucleic acid for each multiplex assay. Multiplex 1 detected influenza virus type A and influenza virus type B using probes for the matrix protein [3,4]. Multiplex 2 detected human coronavirures 229E, NL63, OC43, and HKU1 by using probes from the 1b gene [3], human metapneuoviruses A and B using probes for the N gene [5], and human bocavirus using probes for the NP1 gene [6]. Multiplex 3 detected parainfluenza viruses 1, 2, 3, and 4 using probes for the HN gene [7], adenovirus using probes for the Hexon gene [7], and respiratory syncytial viruses (RSV) A and B using probes for the NC gene [7]. Multiplex 4 detected human rhinovirus using probes for the 5’-untranslated region [8] and the human ribonucleoprotein P gene, included as internal control for the quality control of the sample, extraction, and amplification. For negative samples, results for viruses were considered negative only if amplification of the human ribonucleoprotein P gene was positive. Laboratory procedures to prevent PCR contamination were strictly followed, and positive (purified viral nucleic acids from Vircell, Spain) and negative controls (without sample and/or nucleic acid) were included.

For influenza A-positive samples, a typing RT-PCR assay was performed following the WHO RT-PCR protocol [9]. The assay targets the matrix gene as confirmation control and the HA gene with different probes (influenza A H1N1pdm09, old-seasonal H1N1, and H3N2) for virus typing. For influenza B-positive samples, RT-PCR was performed as described previously [10] for the HA gene to distinguish B/Yamagata and B/Victoria lineages.

### D.I. Ivanovsky Research Institute of Virology (Moscow, Russian Federation)

Real-time RT-PCR was performed using a Ribo-prep kit (E. coli) (AmpliSens, Moscow, Russia) to extract RNA and DNA, a [Reverta-L](http://www.interlabservice.ru/en/catalog/index.php?sid=1037&id=4828) kit (AmpliSens) for reverse transcription, an influenza virus A real-time PCR kit (AmpliSens) to amplify A/H1N1 and H3N2 influenza genes, and an in-house kit for influenza B lineages. Reactions were performed in Rotor Gene 6000 (Corbett Research, Australia) and DTlite Real-Time PCR System (DNA technology, Russia).

### Federal and National Influenza Centre in the Research Institute of Influenza (St. Petersburg)

RNA was isolated from 150–200 μL of Viral Transfer Medium containing two nasal swabs using an AmpliSense RIBO-prep kit or Qiagen RNeasy Mini kit. RT-PCR for influenza A and B viruses was performed using an AmpliSense Influenza virus A/B-FL kit (InterLabService) with reverse transcription with a Reverta-L kit (InterLabService) or using a OneStep RT-PCR Kit (Qiagen) with CDC primers and probes.

Yamagata or Victoria lineage influenza B virus-specific sequences were determined in all influenza B virus-positive specimens using Qiagen OneStep RT-PCR Kit with WHO-recommended primers and probes. Real-time PCR was carried out on a Rotor-Gene 6000 (Corbett Research) or CFX96 Touch Real-Time PCR Detection System (BIO-RAD).

Virus isolation was performed in MDCK cell culture and 10-day-old chicken embryos exposed for 72 h at 34°C according to the approved method [11]. The HI test was performed according to the standard method recommended by the WHO with 0.75% suspension of human red blood cell, group 0, Rh+ for influenza A(H3N2) and B viruses. In the case of influenza A/H1N1pdm09 isolates, 1% suspension of chicken erythrocytes was used [1].

Amplification of cDNA was performed by standard method using the original primers. Sequencing of influenza virus A and B genome fragments (genes HA, NA, M, and NS) was carried out not only for isolated viruses but also for nasal swab specimens on an ABI PRISM 3100-Avant Genetic Analyzer (Applied Biosystems) with a BigDye Terminator Cycle Sequencing Kit v3.1 (Life Technologies).

### National Influenza Reference Laboratory, Istanbul Faculty of Medicine, Turkey

Nasal or nasopharyngeal swabs were collected using Virocult (Medical Wire & Equipment). Total nucleic acid was extracted using an EZ1 Virus mini kit V2.0 (Catalog no. 955134, Qiagen,Germany). A real-time RT-PCR based, multiplex FTD Respiratory Pathogens 21 kit (Fast-track Diagnostics Ltd., Malta) was used for detection of respiratory pathogens on a RotorGene Q platform (Qiagen, Germany). The kit contains an internal control and can detect the following pathogens: influenza A , A(H1N1), and B; rhinovirus; coronavirus NL63, 229E, OC43, and HKU1; parainfluenza 1, 2, 3, and 4; human metapneumovirus A/B; bocavirus; *Mycoplasma pneumoniae*; respiratory syncytial virus A/B; adenovirus; enterovirus; and parechovirus. For detection of influenza H3 subtype and influenza B/Yamagata and B/Victoria lineages, real-time RT-PCR method was performed using an ABI 7500 platform with primers and probes according to the CDC protocol [12].

### References

1. World Health Organization, Manual for the laboratory diagnosis and virological surveillance of influenza, WHO Press, Geneva, 2011.

2. WHO. Influenza. Global Influenza Surveillance and Response System (GISRS) and laboratories (2013) National Influenza Centres. Available at: http://www.who.int/influenza/gisrs_laboratory/national_influenza_centres/list/en/index3.html. Accessed: January 16, 2014.

3. He J, Bose ME, Beck ET, Fan J, Tiwari S et al. Rapid multiplex reverse transcription-PCR typing of influenza A and B virus, and subtyping of influenza A virus into H1, 2, 3, 5, 7, 9, N1 (human), N1 (animal), N2, and N7, including typing of novel swine origin influenza A (H1N1) virus, during the 2009 outbreak in Milwaukee, Wisconsin. J Clin Microbiol 2009; 47:2772–2778.

4. Suwannakarn K, Payungporn S, Chieochansin T, Samransamruajkit R, Amonsin A et al. Typing (A/B) and subtyping (H1/H3/H5) of influenza A viruses by multiplex real-time RT-PCR assays. J Virol Methods 2008; 152:25–31.

5. Kuypers J, Martin ET, Heugel J, Wright N, Morrow R, Englund JA. Clinical disease in children associated with newly described coronavirus subtypes. Pediatrics 2007; 119:e70–e76.

6. Matsuzaki Y, Takashita E, Okamoto M, Mizuta K, Itagaki T et al. Evaluation of a new rapid antigen test using immunochromatography for detection of human metapneumovirus in comparison with real-time PCR assay. J Clin Microbiol 2009; 47:2981–2984.

7. Neske F, Blessing K, Tollmann F, Schubert J, Rethwilm A et al. Real-time PCR for diagnosis of human bocavirus infections and phylogenetic analysis. J ClinMicrobiol 2007; 45: 2116–2122.

8. van de Pol AC, van Loon AM, Wolfs TF, Jansen NJ, Nijhuis M et al. Increased detection of respiratory syncytial virus, influenza viruses, parainfluenza viruses, and adenoviruses with real-time PCR in samples from patients with respiratory symptoms. J Clin Microbiol 2007; 45:2260-2262.

9. WHO(2012) Real-time PCR group protocol #2, WHO molecular diagnosis of influenza virus in humans, November 2012 update.. Available at: http://www.who.int/influenza/gisrs_laboratory/molecular_diagnosis_influenza_virus_humans_update_201211.pdf. Accessed: January 16, 2014.

10. WHO (2011) Real-time PCR group protocol #1, WHO molecular diagnosis of influenza virus in humans. Available at: http://www.who.int/influenza/resources/documents/molecular_diagnosis_influenza_virus_humans_update_201108.pdf. Accessed: January 16, 2014.

11. Sominina A, Burtseva E, Eropkin M, Karpova L, Zarubaev V et al. Influenza surveillance in Russia based on epidemiological and laboratory data for the period from 2005 to 2012. Am J Infect Dis 2013; 9:77–93.

12. CDC (2009) CDC protocol of realtime RTPCR for swine influenza A(H1N1). Available at:<http://www.who.int/csr/resources/publications/swineflu/CDCrealtimeRTPCRprotocol_20090428.pdf>. Accessed: January 23. 2014.
